# Re-assessing the notion(s) of craft standardization through diversity statistics: A pilot study on Late Chalcolithic pottery from Arslantepe in Eastern Anatolia

**DOI:** 10.1371/journal.pone.0245660

**Published:** 2021-01-20

**Authors:** Pamela Fragnoli

**Affiliations:** Department for Archaeometry, Austrian Archaeological Institute, Austrian Academy of Sciences, Vienna, Austria; University at Buffalo - The State University of New York, UNITED STATES

## Abstract

This paper proposes a new range of diversity indexes applicable to ceramic petrographic and geochemical data and potentially to any archaeological data of both metric and non-metric nature in order to assess the degree of craft standardization. The case study is the Late Chalcolithic pottery from Arslantepe in eastern Anatolia, ideal to test the standardization hypothesis, i.e. the assumed correspondence between craft standardization and increased rates of production, which in turn correlate with economic specialization. The results suggest that the procurement and processing of raw materials are more sensible indicators of standardization than vessel shape variability. Higher standardization is connected with the scale of production rather than with the use of the wheel or its rotational speed. The socio-economic centralization marks a process of labor division within the operational sequence and, more generally, a shift from communal to more segregated potting practices. As a result, the variability of both technical procedures and end products increases. In contrast univocal trends towards standardization can be found in coeval contexts from northern Mesopotamia, where the incipient urbanization served to create bonds between vessel makers, favoring the transmission of models and practices regardless of the centralized power.

## Introduction

Standardization is commonly perceived as a process of reduction in artifact variability at several levels: raw materials composition, manufacturing techniques, forms and dimensions as well as decorations. The standardization of products is generally assumed to be the result of a higher rate of production that typically characterizes the economic organization of early complex societies [1–10]. The surplus centralized by the elites allowed some individuals to be exempted from the primary production and focus more intensively on craft activities in exchange for food. This enhanced the routinization and mechanization of gestures that was reflected in an increased homogenization of finished products [3, 11, 12]. Therefore, the increased standardization has been often viewed as indicating the activity of specialized artisans. However, the relationship between artifact standardization and craft specialization is far from linear and has been called into question by several ethnoarchaeological studies [3, 10, 13–18]. In pottery production, increased levels of standardization and specialization are commonly associated with the introduction of rotating devices in the manufacturing process. On the one hand, this technological innovation required the acquisition of specific motor skills through long apprenticeship and continuous practice and, on the other hand, it favored the repetitiveness of gestures and enhanced production times and rates [19–21].

So far, standardization studies on archaeological ceramics have mainly focused on measuring the vessels’ dimensional variation through a sophisticated range of measures [5, 18, 22–28], while non-metric attributes, such as typological and technological attributes, have received less attention [however, see 29–34]. In the last two decades the assessment of compositional variability has gained importance, but the integration between petrographic and geochemical data as well as the correlation with morphological, dimensional and technological variables need to be further explored [31, 33–39].

This paper intends to exploit the potential of compositional analyses for assessing craft specialization and artifacts’ standardization. The case study is the Late Chalcolithic (ca. 4700–3200 BCE cal.) pottery assemblage from Arslantepe in eastern Anatolia, ideal to test the standardization hypothesis. The standardization hypothesis proposes that more uniformity in the vessel assemblages is due to higher rates of production, which create task mechanization and routinization (i.e. motor habits) [3–6, 11, 27]. Many scholars consider craft standardization as evidence of specialization, thus as a key aspect in the political economy of complex societies [2, 36, 40]. As argued by Hilditch [33], craft standardization has been frequently seen as the result of a unilinear process intensified by the introduction of the potter’s wheel that enhanced both time and scale of production; however, little attention has been dedicated to single variations along the *chaîne opératoire* to assess where and how standardized gestures and behaviors appear.

In his paper “Does the standardization of ceramic pastes really mean specialization?” Arnold claimed that paste composition provides information primarily on the geological context rather than on the production organization [41]. His assumption was based on geochemical data of ceramic vessels produced at a household level from different ethnographic communities in Mexico, Peru and Guatemala. The present paper demonstrates instead that the variations in paste recipes can be used as indicators of production organization at least at an intra-site level. To achieve this aim, different compositional analyses—i.e. bulk geochemistry and thin section petrography—have to be integrated with selected technological and typological features. Interpretations in terms of production organization are further favored in cases of variegated pottery assemblages related to distinct levels of specialization and produced over a long time span marked by drastic socio-economic changes.

The aim of this paper is to assess whether the gradual process of economic centralization that led to the formation of an early state society by the end of the 4th millennium BCE at the site of Arslantepe (Malatya, Turkey) implied the homogenization and increased standardization of pottery production and, in particular, of the raw material procurement patterns and paste preparation modes. To this end, petrographic and geochemical data of locally-produced vessels are elaborated using procedures borrowed from diversity statistics. Finally, the trends identified are compared with vessel shape variability, manufacturing techniques and production rates, in order to detect differences and correlations in technological variations within the various steps of the *chaîne opératoire*.

## Economic centralization, technical innovation and production serialization at Late Chalcolithic Arslantepe

**Fig 1:**
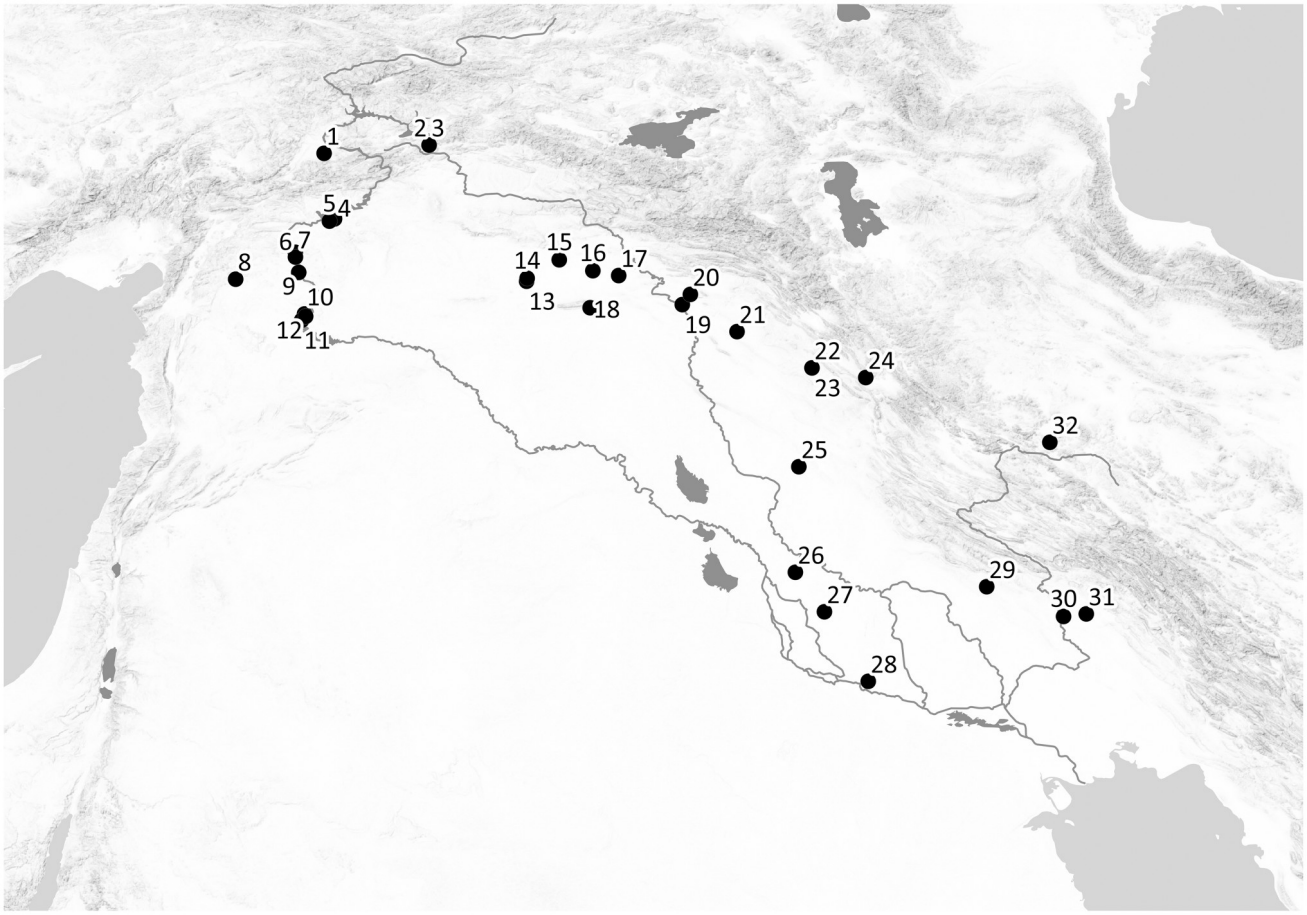
Location of the main investigated Late Chalcolithic sites in greater Mesopotamia. 1. Arslantepe; 2. Tepecik; 3. Norşuntepe; 4. Samsat; 5. Kurban Höyük; 6. Hacınebi Tepe; 7. Zeytinli Bahçe Höyük; 8. Oylum Höyük; 9. Jerablus Tahtani; 10. Jebel Aruda; 11. Habuba Kabira South; 12. Tell Sheikh Hassan; 13. Tell Brak; 14. Tell Feres al-Sharqi; 15. Tell Leilan; 16. Tell Hamoukar; 17. Tell el-Hawa; 18. Grai Resh; 19. Nineveh; 20. Tepe Gawra; 21. Surezha; 22. Logardan; 23. Girdi Qala; 24. Gerdi Resh; 25. Tell Rubeidheh and Tell Hassan; 26. Tell Uqair; 27. Abu Salabikh; 28. Uruk; 29. Teppe Farukhabad; 30. Susa; 31. Chogha Mish; 32. Godin Tepe. Map: M. Karaucak through the topographic data courtesy of Shuttle Radar Topography Mission (SRTM), DOI:/10.5066/F7K072R7.

Arslantepe is a multi-layered settlement located in the Malatya Plain in Eastern Anatolia, a few kilometers south of the Euphrates River and on the northern side of the Anti-Taurus Mountains (Fig 1). The Late Chalcolithic phases reveal the site’s historical relevance in the formation process of early-state societies and the emergence of social and economic inequality [42–45]. During the Late Chalcolithic period all Mesopotamia and related regions—including the upper courses of the Euphrates and Tigris in Anatolia, the Trans-Tigridian regions, and the Amuq and Susiana plains—share structural changes in the economic and political organization of the communities. These results in the emergence of complex societies characterized by political hierarchies, economic centralization and, in many areas, the first urban centers [45, 46].

**Fig 2:**
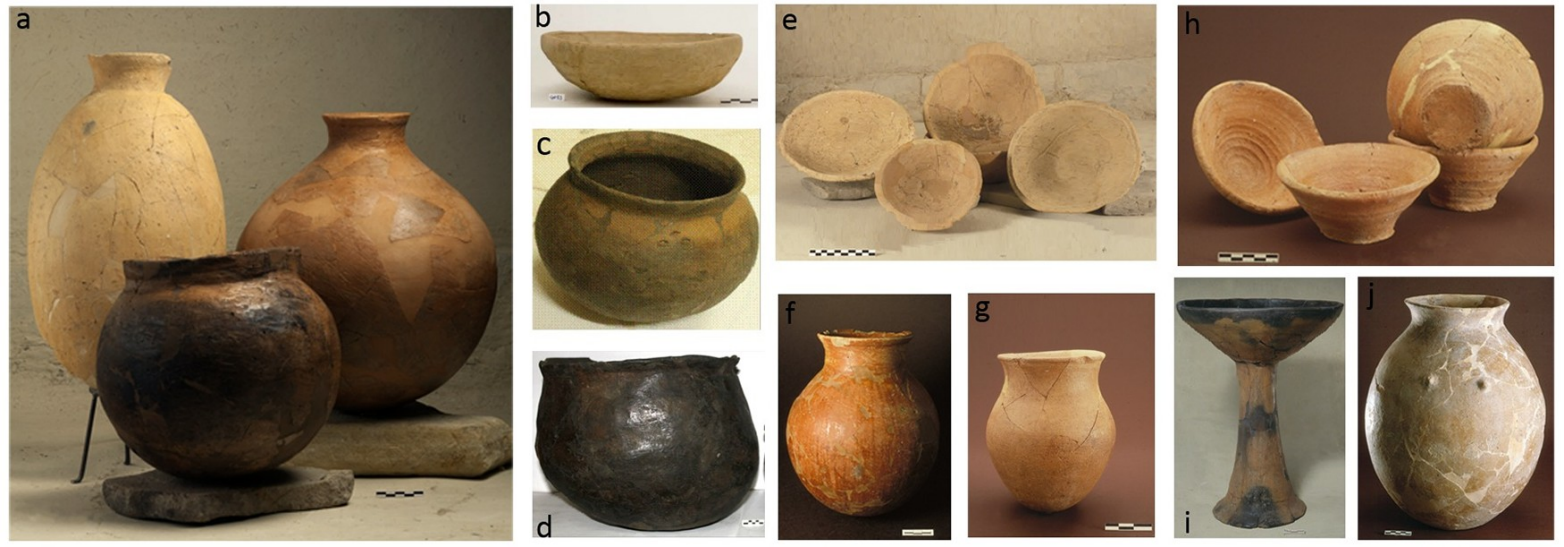
Examples of LC pottery from Arslantepe. a. LC1-2 storage jars and kitchen wares; b. LC1-2 handmade Coba Bowl; c. LC3-4 wheel-finished cooking pot; LC3-4 handmade cooking pot; e. LC3-4 wheel-finished mass-produced bowls; f. LC3-4 wheel-finished red-slipped ware (RIB) small jar; g. LC5 wheel-made light-colored fine ware jarlet; h. LC5 wheel-made light-colored coarse ware mass-produced bowls; i. LC5 handmade red-black burnished ware high-stemmed bowl; j. LC5 handmade kitchen ware and storage jar. Image: Archive of the Missione Archeologica Italiana in Anatolia Orientale (MAIAO).

The rich Late Chalcolithic pottery assemblages of Arslantepe, all found in primary contexts, refer to various spheres of pottery production and manufacturing traditions, and provide a significant record to investigate organizational, economic, and cultural changes. The Late Chalcolithic sequence is divided into three main phases corresponding to the Late Chalcolithic 1–2, Late Chalcolithic 3–4 and Late Chalcolithic 5 in the Mesopotamian chronology [46, 47]. The first Late Chalcolithic phase (LC1-2 or Arslantepe period VIII in the site sequence: ca. 4700–3900 BCE) consists in eight levels excavated so far; all are characterized by small domestic units, typically with some rooms devoted to food processing [48, 49]. The pottery is entirely handmade throughout the whole period, with surfaces either scraped or left plain, while burnishing and slipping rarely occur among surface treatments (Fig 2a and 2b). As for shapes, bowls predominate over beakers, basins, bottles, jars, and *pithoi*. Approximately 15% of the pottery is mass-produced (Fig 2b), namely light-colored coarse chaff-tempered bowls with scraped bottoms generally referred to as “Coba bowls” [50]. In the pottery assemblages of all Mesopotamia this period marks the disappearance of painted decorations and high-fired fine grit fabrics, testifying to a new role of ceramic containers within the communities [30, 48]. Pottery production loses its symbolic and representative character and becomes oriented towards efficiency, functional goals and serialization. These changes are related to increasingly repetitive and more and more widely shared social practices such as food consumption and redistribution.

Increasing social complexity at Arslantepe is more clearly visible in the subsequent Late Chalcolithic phases. During the Late LC3-4 (period VII: ca. 3900–3400 BCE), the settlement enlarges and becomes internally structured in residential and public areas [44]. Two large tripartite buildings occupied the uppermost part of the hill; their monumentality and decorations together with the thousands of clay sealings and mass-produced bowls (Fig 2e) found in them have been interpreted as evidence of ritualized redistributive activities [45: 8–10, 51]. This phase marks also the introduction of rotating devices in the ceramic manufacturing process. In addition to the wheel-finished mass-produced bowls, the pottery assemblage comprises wheel-finished plain or red-slipped burnished jarlets, beakers and jars as well as handmade and wheel-finished globular cooking pots [52, 53] (Fig 2c, 2d and 2f). The occurrence of marks on some wheel-finished vessels has been interpreted as a means for the producers to recognize their own pots in shared drying areas and firing facilities [54, 55]. At the end of the period, a few handmade red-black or monochrome burnished vessels—mainly high-stemmed bowls—of Central-Anatolian influence appeared [56], and this coincides with the first attestation at the site of a caprine-oriented husbandry strategy [57].

During the final phase of the Late Chalcolithic (LC5, Arslantepe period VIA: ca. 3400–3200 BCE) the centralization of resources progressed and a local ‘early state’ society with a proto-palatial complex was established at the site [42, 44, 58–62]. The mass-production of bowls (Fig 2h) devoted to the redistribution of meals increased due also to the hypothesized introduction of the fast wheel in the manufacturing process, and potter’s marks totally disappeared. The rest of the ceramic repertoire (Fig 2g and 2j) comprises wheel-finished light-colored jars, jarlets and high-stemmed bowls, as well as handmade storage containers and cooking pots [62–65]. The handmade red-black and monochrome burnished vessels (Fig 2i) increase in number and now exhibit a wider formal and functional repertoire including bowls, cups, jars, jarlets, typical high-stemmed bowls and a few *pithoi* [56, 62, 66–68].

## Wares, forming techniques and morphometric analyses

At Arslantepe ceramic wares have been conventionally distinguished since the 1970s on the basis of specific macroscopic hierarchical criteria, namely texture (coarse/semifine/fine), tempering material (chaff/grit/mixed), shaping techniques (handmade/wheel-finished), surface treatments (slipping/burnishing/smoothing) and colors (red-black/black/red/brown/light-colored) [52, 62, 64, 65]. Morphological criteria have been considered separately, at another level of analysis, and formed the basis for further functional observations. This classification statistically consolidated across decades thanks to the analysis of thousands of diagnostic sherds and complete vessels found in primary contexts of deposition [48, 49, 62, 64]. Interestingly, the correlation between shapes (morphological types) and wares increases through time. It is in fact during the LC5 that the strongest correspondence between pots with a specific shape and wares occurs, with only two exceptions: the high-stemmed bowls (Fig 2i) and small jarlets with an S-shaped/sinuous profile (Fig 2g), both realized in fine light-colored wheel-finished and red-black burnished ware. In the previous LC3-4 period most vessel shapes are invariably realized in either wheel-finished or handmade wares, the former being anyway a minority of the total assemblage [69]. The term “mass-produced”, conventionally adopted in Mesopotamian Archaeology, refers to specific categories of bowls produced on a large scale—usually hundreds or even thousands of items of the same vessel category in terms of shape, function, and approximate size—and found all together in the same contexts. This term therefore crosses technical, quantitative and typological criteria.

**Fig 3:**
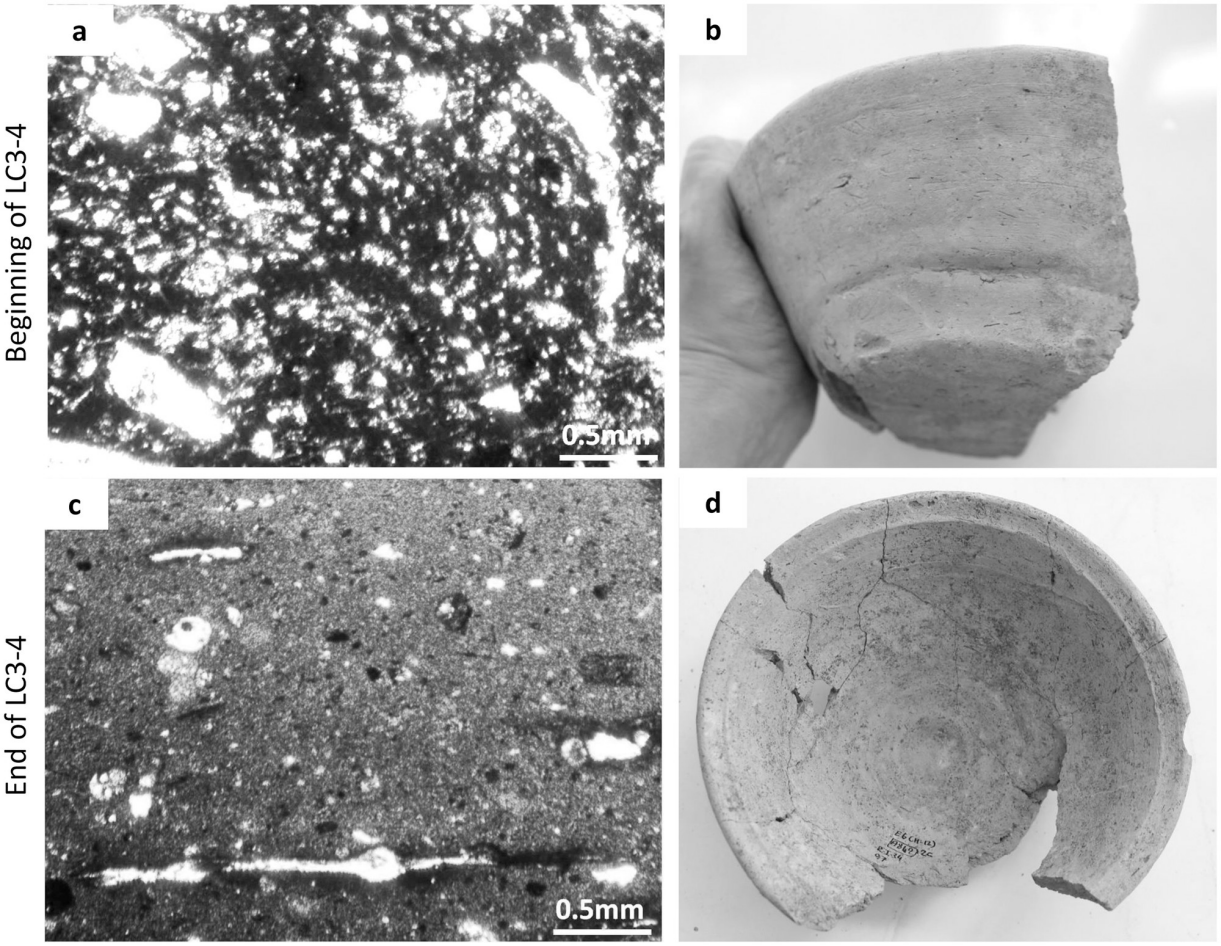
Microscopic and macroscopic features of mass-produced bowls at the beginning and end of the LC3-4 phase, evidencing a diachronic increase in the use of the rotational kinetic energy. Image: ÖAW-ÖAI / P. Fragnoli.

In the late 1960s and 1970s, Alba Palmieri already argued for the introduction and frequent use of rotating devices in the manufacture of LC3-4 pottery [70] and the introduction of the fast-wheel by the LC5 due to the recurrence on some vessel shapes of inner concentric grooves and underside string cut impressions [71]. Palmieri’s initial observations were then confirmed and broadened by other scholars working on the LC material from Arslantepe [48, 52, 62, 64, 69]. I cannot discuss this hypothesis in detail here, but following the more recent contributions on wheel-based forming techniques [72] I am currently investigating the LC repertoire. My recent work demonstrates that during the LC4 (end of period VII in the site sequence) the use of turning devices consolidates by entering progressively earlier stages of the forming sequence [73, 74]. This is especially evident for the mass-produced bowls at both a microscopic and macroscopic level (Fig 3). Microscopically, the temper fraction follows strongly oriented patterns and the clay matrix shows evidence of shear stresses. Macroscopically, concentric striations/grooves spread along the entire vessel profiles, the wall thickness gets gradually thinner towards the rim, profiles gain in symmetry, while linear discontinuities and anomalies in correspondence of structural joints decrease or even disappear.

**Fig 4:**
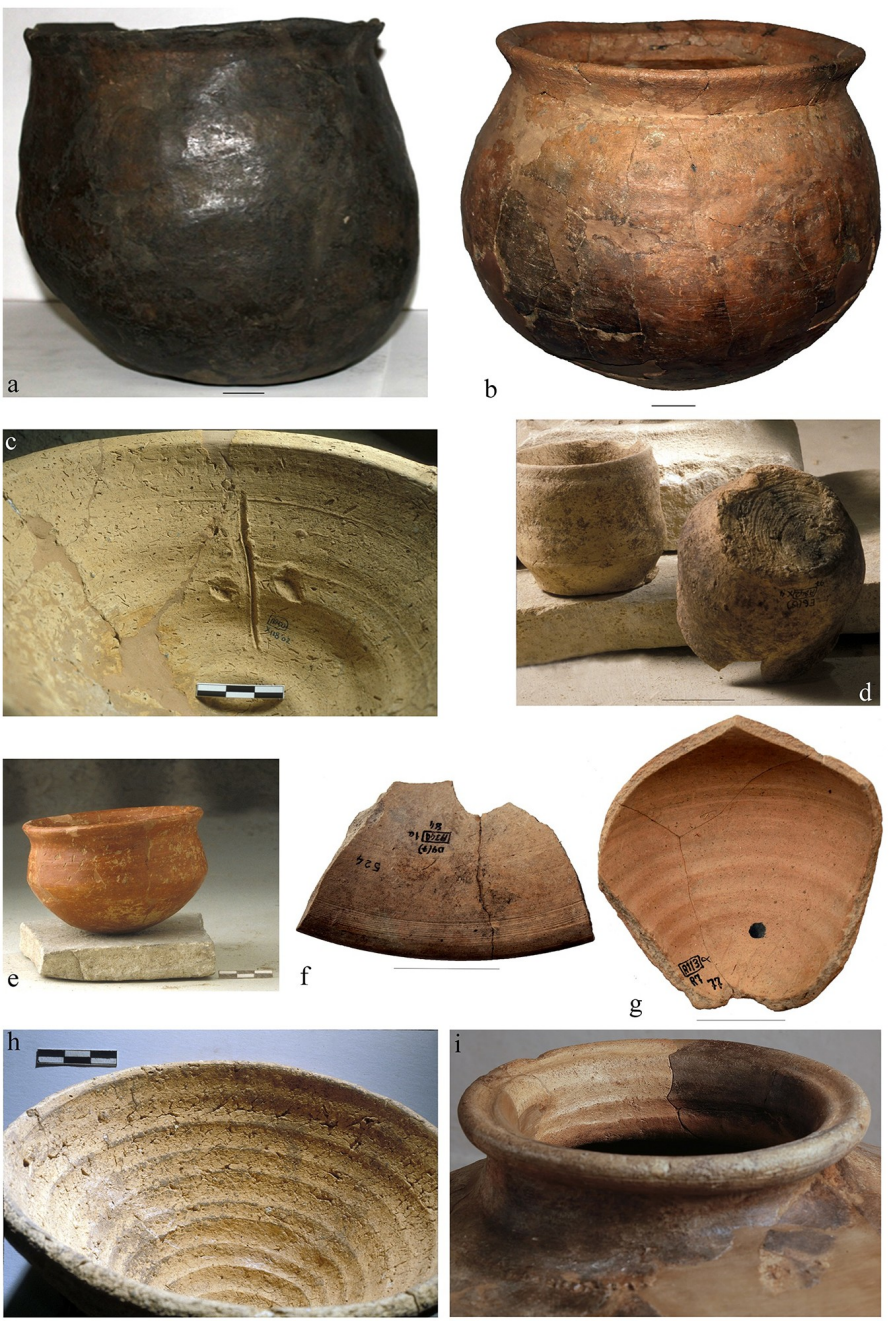
a. LC3-4 handmade kitchen ware; b. LC3-4 wheel-finished kitchen ware; c. LC3-4 wheel-finished mass-produced bowl with potter’s mark; d. LC3-4 wheel-finished chaff-tempered smoothed ware; e. LC3-4 wheel-finished red-slipped burnished ware; f. LC 5 wheel-finished light-colored fine ware (internal side of the foot of a high-stemmed bowl; g. wheel-finished light-colored fine ware (internal side of a jarlet); h. LC5 wheel-finished mass-produced bowl; i. neck of a LC5 wheel-finished light-colored semifine ware large jar. Image: Archive of the Missione Archeologica Italiana in Anatolia Orientale (MAIAO).

**Fig 5:**
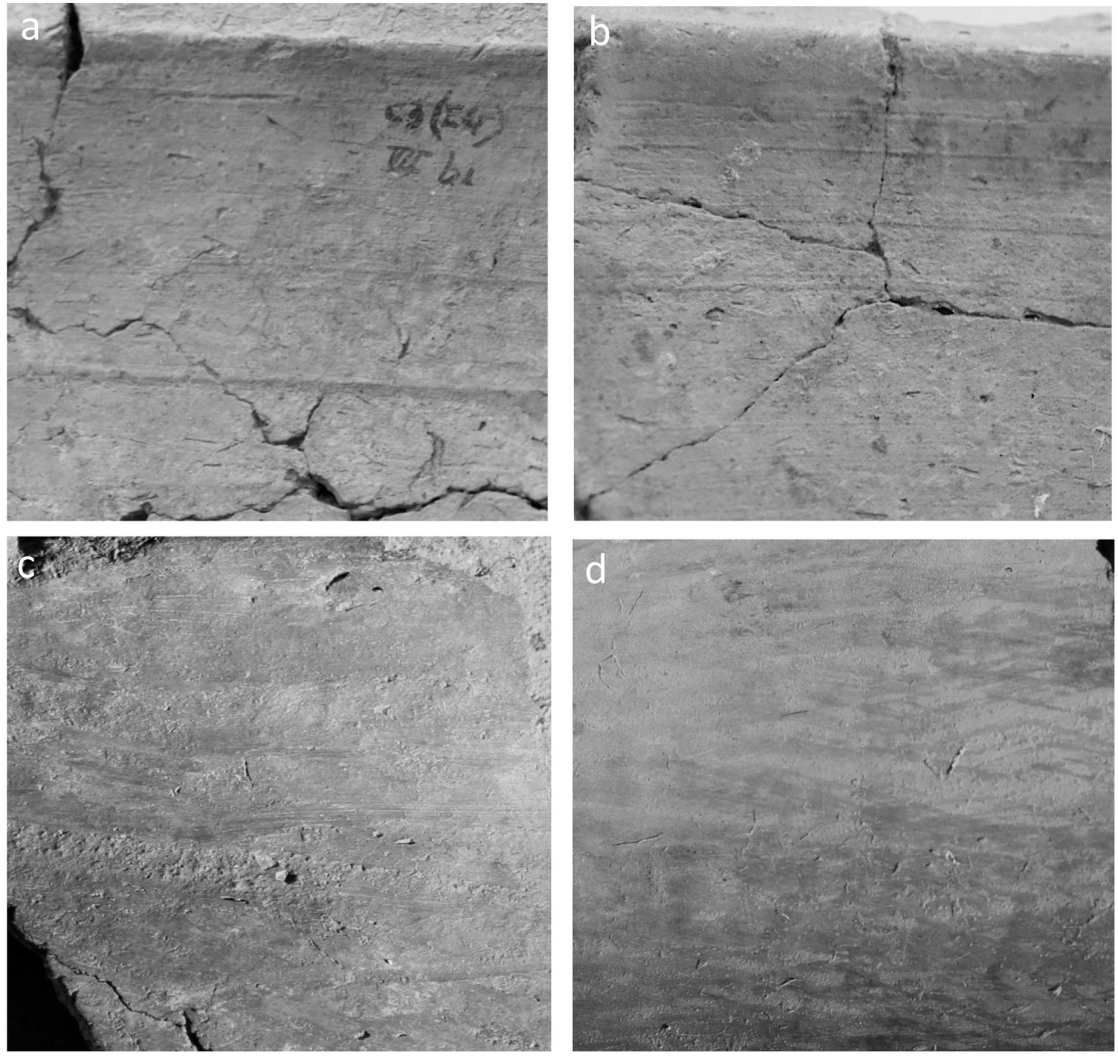
Striations occurring with (a-b) or without (c-d) the use of rotating devices. Image: ÖAW-ÖAI / P. Fragnoli.

In this paper vessels were distinguished depending on whether or not they were produced with the help of rotating devices, whatever the stage of the forming sequence these devices entered in. These two large categories are here referred to as handmade and wheel-finished vessels, even though the latter might have combined different forming techniques. This broad categorization puts the emphasis on the most significant technical innovation of the period, i.e. the introduction of turning devices, and related hypotheses on craft specialization and standardization. At Arslantepe wheel-finished vessels are mainly distinguished by horizontal and parallel striations or grooves that might appear on the different surfaces of the vessel body (Fig 4). These diagnostic traces result from finishing, thinning, shaping or cutting vessels while turning. Striations might also occur on vessel surfaces without the use of any rotating devices due to finishing procedures like smoothing and burnishing. However, striations visibly differ depending on whether or not they were generated by the application of the rotational kinetic energy (Fig 5). On wheel-finished vessels striations appear as dense, fine, ribbed, continuous and homogeneous lines, which are evenly spaced from each other and organized in horizontal parallel concentric bands. Moreover, a typical fluidized surface microtopography is often associated with these features. The striations obtained without the rotational kinetic energy are instead much more heterogeneous both in shape and orientation [72: 236–240]. Further diagnostic features of wheel-finished vessels are regular wall thicknesses, stretched surfaces and strong symmetry of profiles.

To assess the morphological variability of the LC3-4 to LC5 pottery repertoire, Guarino and D’Anna calculated the coefficient of variation (CV) on the ratios between maximum diameter and height, rim diameter and maximum diameter, and rim diameter and height of specific vessel types [66, 71]. Usually, an assemblage of ceramics with CV below 10% is considered to have a low level of variability as the result of specialized potters [5, 18, 22, 27]. At Arslantepe most of the LC3-5 vessels present higher CVs (Table 1). Values indicating a higher standardization surprisingly recur in the handmade vessels, while the serial production of bowls with the help of rotating devices does not inevitably imply a decreased variability. Lastly, the LC5 does not mark an increase in standardization despite the stronger incidence of the rotational kinetic energy in the manufacturing process.

**Table 1:** Coefficient of variations (CV) calculated on LC3-4 to LC5 classes of vessels [64, 69].

	Manufacturing	Vessel classes	Considered ratios	CV ranges
**LC3-4**	wheel-finished	serving/storage jars	Ø rim / Ø max	9.37–21.30
		cooking pots	Ø rim / Ø max	8.44–9.87
			Ø rim / height	10.94–17.87
		mass-produced bowls	Ø rim / height	9.57
	handmade	serving/storage jars	Ø rim / Ø max	17.59
		cooking pots	Ø rim / Ø max	5.94–6.82
**LC5**	wheel-finished	mass-produced bowls	Ø rim / height	13.06
		necked-jars	Ø max / height	4.6–10.5
			Ø rim / Ø max	13.1–17.8
			Ø rim / height	11.9–22
		fine jarlets	Ø rim / height	10.4–13.2
			Ø rim / Ø max	10.7–11.7
	handmade	cooking pots	Ø max / height	6.8–13.2
			Ø rim / Ø max	5.1–16.5
		monochrome/red-black burnished ware	Ø rim / height	5.15–17.58

## Geological setting and raw material supply

**Fig 6:**
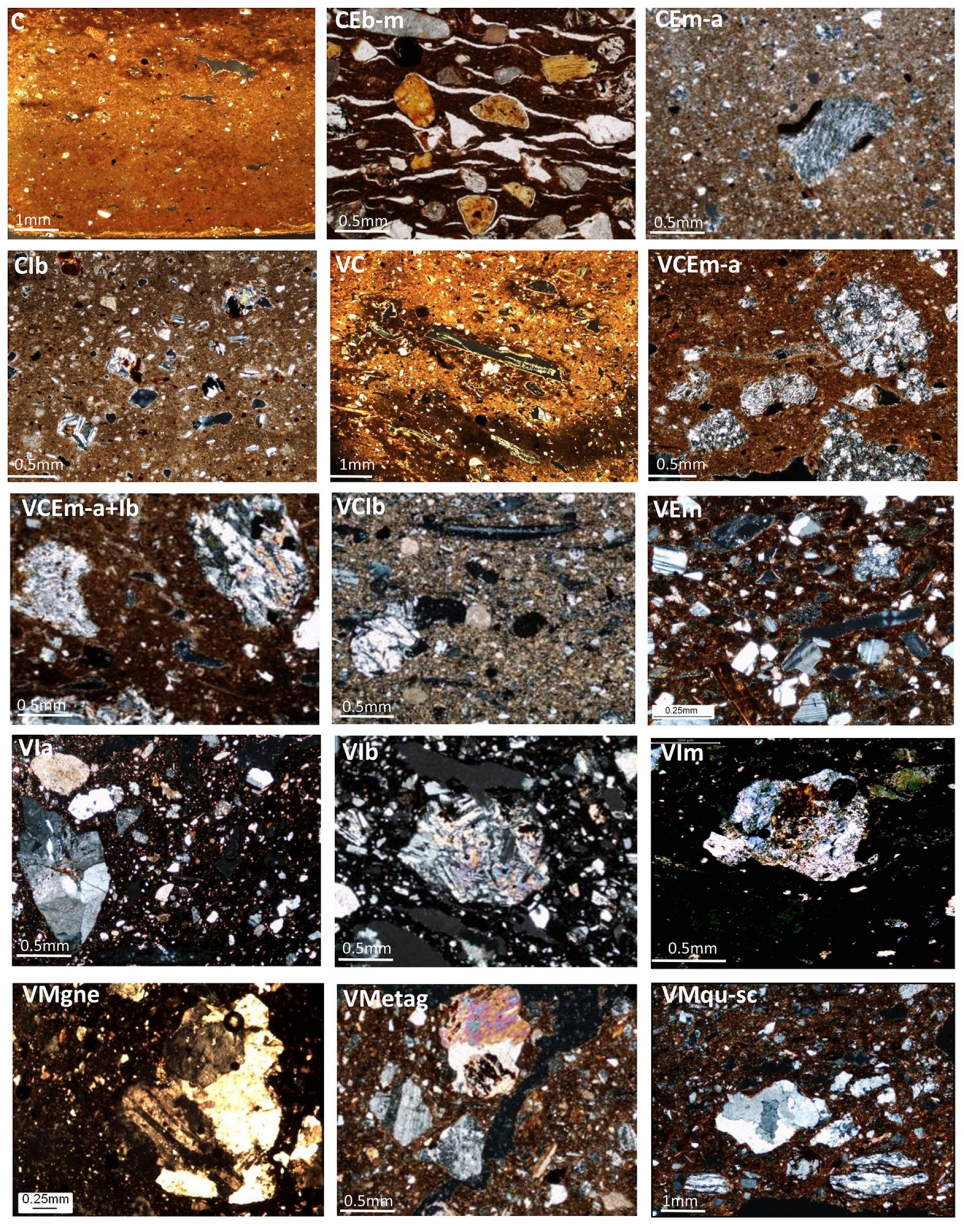
Selected micro-pictures illustrating the main petro-groups. Image: ÖAW-ÖAI / P. Fragnoli.

The site of Arslantepe (Fig 6) lies on Miocene lake sediments, mainly consisting of calcareous clays, limestones and sandstones [75]. Immediately northeast of the site, at a distance of 700 m, is the remnant of the Middle Miocene Orduzu volcanic suite [76] composed of rhyolites, trachyandesites, basaltic trachyandesites and quartz-micromonzonites [77]. Approximately 5.5 km further east we find the Late Cretaceous Baskil magmatics and the Maastrichtian to the Early Eocene Yüksekova/Elazığ complex, dominated by volcanic and intrusive rocks ranging from mafic to felsic affinities, i.e. gabbros, diorites, tonalities, monzonites, basaltic andesites, andesites, dacites and rhyolites [78, 79].

More distant and spatially widespread are the units of the Antitaurus mountain chains that start rising 7 to 10 km south of the site. The western part of these units belongs to the Malatya metamorphics distinguished by Carboniferous to Triassic meta-carbonate rocks, mica schists, phyllites, slates, meta-clastic rocks and meta-cherts [80, 81]. The eastern part is instead dominated by the Late Cretaceous Ispendere ophiolites and the Middle Eocene Maden Complex. The former exhibit an intact ophiolitic sequence intruded by granites [82], the latter a volcanosedimentary sequence with conglomerates, sandstones, limestones, mudstones, spilitic lavas, radiolarites, cherts, altered basalts and andesites [80, 81, 83].

Most of the above-mentioned formations were exploited for producing vessels at Arslantepe, with distinct patterns according to the chronological phases and/or type of wares [84–86]. The variety of geological formations locally available [87] represents a double-edged sword from a methodological point of view and especially for minero-petrographic applications. On the one hand, we are able to outline precise strategies of raw material procurement within the local landscape; on the other, we often have difficulties in distinguishing local from imported vessels. To this end, thin section petrography is integrated with geochemical analyses of both vessels and local raw materials [84–86].

## Sampling strategy and methods

The samples under investigation represent the variety of ceramic shapes and wares produced at the site along the entire Late Chalcolithic sequence (ca. 4700–3200 BCE). As illustrated above, within the assemblage of each period, wares have been macroscopically identified on the basis of the consistent co-occurrence of fabrics, manufacturing techniques, surface treatments, firing procedures, and, when present, decorations. Sampling strategies aimed at accounting for the duration of each period and the associated amount of materials recovered so far. This allows us to mitigate the cumulative blurring effect, namely the higher variability that production events generate along longer time-spans [36]. Thus, mostly represented here is the vast vessel repertoire of the long-lasting LC3-4 phase (97 samples). By contrast, the few samples (19) from the LC1-2 refer to a single context within the entire phase and are rather intended to act as reference for a non-standardized production [48, 49]. The assemblages of the following LC3-4 and LC5 phases (51 samples)—which provide us with evidence of economic centralization, intensification of production rates and introduction of the wheel—are instead those used in this paper to test the standardization hypothesis. At any rate, this study is intended as a first small-scale experiment aimed at testing the potential of diversity statistics in assessing craft standardization with the objective of being subsequently applied and adjusted to a wider sampling also including other geographic and chronological frameworks. The permission for pottery sampling and-analysis was kindly issued by the Turkish authorities.

Since the paper aims at assessing the uniformity of the local production modes, vessels of underrepresented foreign typology (e.g. the rare beveled rim bowls found at the site) or not matching geochemically and petrographically with local reference fields have been excluded [74, 84, 85]. The petrographic data used in this paper refer to 167 thin sections (Tables 2 and 3; Fig 6) that are grouped according to: 1) calcareous versus non-calcareous clay matrix; 2) the presence/absence of organic temper; 3) the geological origin of mineral and rock inclusions, which may refer to variegated volcanic, plutonic and metamorphic environments. Based on petrographic groupings, 60 representative samples were selected to be analyzed through wavelength-dispersive X-ray fluorescence (Table 5). Measurements were undertaken at the Archea Laboratory in Warsaw using the wavelength dispersive X-Ray Fluorescence spectrometer PANnalytical AXIOS. After being ignited at 900°C, 1.5-2g powder of each sample was melted with a lithium-borate mixture and cast into small discs. Major elements were normalized to a constant sum of 100% and trace elements under the detection limit (e.g. Y, Pb, Nb, Cu) were removed. Detailed descriptions of the petro-groups as well as “more traditional” bivariate and multivariate statistical elaborations of geochemical data have already been published in the contributions of the author indicated above and for this reason are not reported again here in detail. Petrography has been applied to a higher number of samples, since it has repeatedly proven to be a more eloquent indicator of local technological practices due to the coarseness of the vessels and the occurrence of variegated and well-delimited geological formations all around the site. The selected petrographic and geochemical data considered here cover the entire local spectrum, which was previously assessed in a wider sampling and along a longer chronological span. The assessment of the diversity parameters proposed in this paper does not require any particular statistical software as they can be easily performed on Excel (S1–S3 Tables).

**Table 2:** List of the samples analyzed petrographically and related petrographic groups.

Sample	Phase	Ceramic ware	Petro-group
126/14*	LC1-2	handmade plain grit ware	NC
127/14*		handmade plain grit ware	CIb
128/14*		handmade plain grit ware	NC
129/14*		handmade plain grit ware	CIb
130/14*		handmade plain ware	VIm
131/14*		handmade plain ware	NC
132/14*		handmade plain ware	NC
133/14*		handmade plain ware	VIa
134/14*		handmade burnished ware	NC
135/14*		handmade burnished ware	NC
136/14*		handmade burnished ware	NC
137/14*		handmade burnished ware	NC
138/14*		handmade burnished ware	VIm
139/14*		handmade mass-produced bowl	VIm
140/14*		handmade mass-produced bowl	NC
141/14*		handmade mass-produced bowl	VIm
142/14*		handmade plain ware	VIa
143/14*		handmade mass-produced bowl	NC
144/14*		handmade mass-produced bowl	NC
3638	LC3-4	wheel-finished red-slipped burnished ware	VCEm-a
3639		wheel-finished red-slipped burnished ware	VCEm-a
3641		wheel-finished red-slipped burnished ware	VCEm-a
3642		wheel-finished red-slipped burnished ware	VCEm-a
3643		wheel-finished red-slipped burnished ware	VCEm-a
3644		wheel-finished red-slipped burnished ware	VCIb
3645		wheel-finished red-slipped burnished ware	VCIb
3646		wheel-finished kitchen ware	VCIb
3647		wheel-finished kitchen ware	VCIb
3648		wheel-finished kitchen ware	VCEm-a+Ib
3649		wheel-finished red-slipped burnished ware	VCIb
3650		wheel-finished chaff-tempered smoothed ware	VCIb
3651		wheel-finished chaff-tempered smoothed ware	VCIb
3654		wheel-finished kitchen ware	VIb
3655		handmade kitchen ware	VIb
3656		wheel-finished mass-produced bowl	VCEm-a
3657		wheel-finished mass-produced bowl	VCIb
3658		wheel-finished mass-produced bowl	VCEm-a
3660		wheel-finished mass-produced bowl	VCEm-a
3661		wheel-finished mass-produced bowl	VCEm-a
3662		wheel-finished mass-produced bowl	VCEm-a
3673		wheel-finished red-slipped burnished ware	VCEm-a+Ib
3674		wheel-finished red-slipped burnished ware	VCEm-a+Ib
3675		wheel-finished red-slipped burnished ware	VCIb
3676		wheel-finished red-slipped burnished ware	VCEm-a+Ib
103/14		wheel-finished chaff-tempered smoothed ware	VCIb
104/14		wheel-finished red-slipped burnished ware	NC
105/14		wheel-finished chaff-tempered smoothed ware	VIb
107/14		wheel-finished chaff-tempered smoothed ware	VIb
159/14		wheel-finished red-slipped burnished ware	VC
257/14		wheel-finished chaff-tempered smoothed ware	VCEm-a+Ib
271/14		wheel-finished red-slipped burnished ware	VCEm-a+Ib
272/14		handmade light-colored ware	VC
273/14		handmade light-colored ware	VIb
274/14		handmade red-black/monochrome burnished ware	NC
275/14		handmade light-colored ware	NC
276/14		handmade kitchen ware	VIb
277/14		handmade kitchen ware	NC
300/14		wheel-finished red-slipped burnished ware	VCEm-a+Ib
301/14		wheel-finished mass-produced bowl	VCEm-a
304/14		wheel-finished mass-produced bowl	VC
305/14		wheel-finished red-slipped burnished ware	VCIb
307/14		wheel-finished kitchen ware	VMgne
309/14		wheel-finished chaff-tempered smoothed ware	VIb
370/14		wheel-finished chaff-tempered smoothed ware	VMgne
371/14		wheel-finished chaff-tempered smoothed ware	VCEm-a+Ib
372/14		wheel-finished chaff-tempered smoothed ware	VIb
375/14		wheel-finished red-slipped burnished ware	VCEm-a+Ib
376/14		wheel-finished red-slipped burnished ware	VC
102/14		wheel-finished red-slipped burnished ware	NC
106/14		wheel-finished chaff-tempered smoothed ware	VCEm-a+Ib
108/14		wheel-finished mass-produced bowl	VC
109/14		wheel-finished mass-produced bowl	VCEm-a
155/14		wheel-finished red-slipped burnished ware	VCEm-a
156/14		wheel-finished red-slipped burnished ware	VCIb
157/14		wheel-finished red-slipped burnished ware	VCEm-a+Ib
158/14		wheel-finished red-slipped burnished ware	VC
160/14		wheel-finished red-slipped burnished ware	VCIb
161/14		wheel-finished red-slipped burnished ware	VCEm-a
162/14		wheel-finished red-slipped burnished ware	VC
163/14		wheel-finished red-slipped burnished ware	VCEm-a
258/14		wheel-finished chaff-tempered smoothed ware	VCIb
259/14		wheel-finished kitchen ware	VIb
260/14		wheel-finished chaff-tempered smoothed ware	VIb
261/14		wheel-finished kitchen ware	VEm
262/14		handmade kitchen ware	VEm
278/14		handmade kitchen ware	NC
279/14		handmade light-colored ware	VCEm-a+Ib
280/14		handmade kitchen ware	VMqu-sc
281/14		wheel-finished chaff-tempered smoothed ware	VCEm-a+Ib
282/14		wheel-finished light-colored fine ware	C
283/14		wheel-finished light-colored fine ware	C
284/14		wheel-finished mass-produced bowl	VC
285/14		wheel-finished light-colored fine ware	C
286/14		wheel-finished light-colored fine ware	C
287/14		wheel-finished light-colored fine ware	VCEm-a+Ib
288/14		wheel-finished light-colored fine ware	NC
289/14		wheel-finished light-colored fine ware	C
290/14		handmade red-black/monochrome burnished ware	NC
291/14		handmade red-black/monochrome burnished ware	NC
293/14		handmade red-black/monochrome burnished ware	NC
294/14		handmade red-black/monochrome burnished ware	NC
295/14		handmade red-black/monochrome burnished ware	NC
296/14		handmade red-black/monochrome burnished ware	NC
299/14		wheel-finished chaff-tempered smoothed ware	VEm
302/14		wheel-finished mass-produced bowl	VCEm-a
303/14		wheel-finished mass-produced bowl	VC
306/14		wheel-finished chaff-tempered smoothed ware	VC
308/14		wheel-finished red-slipped burnished ware	VCIb
366/14		wheel-finished red-slipped burnished ware	VCEm-a+Ib
367/14		handmade red-black/monochrome burnished ware	NC
368/14		wheel-finished red-slipped burnished ware	VCEm-a
369/14		handmade light-colored ware	VC
373/14		handmade kitchen ware	VMqu-sc
374/14		wheel-finished chaff-tempered smoothed ware	VCEm-a+Ib
450/14		wheel-finished light-colored fine ware	NC
310/14		wheel-finished red-slipped burnished ware	VCEm-a
3595	LC5	handmade red-black/monochrome burnished ware	NC
3558		handmade red-black/monochrome burnished ware	NC
3560		handmade red-black/monochrome burnished ware	NC
3594		handmade red-black/monochrome burnished ware	NC
3554		wheel-finished mass-produced bowl	VCEm-a
223/14		handmade red-black/monochrome burnished ware	NC
225/14		handmade red-black/monochrome burnished ware	NC
227/14		handmade red-black/monochrome burnished ware	NC
230/14		handmade red-black/monochrome burnished ware	NC
232/14		handmade red-black/monochrome burnished ware	NC
2/2015		handmade red-black/monochrome burnished ware	NC
4/2015		handmade red-black/monochrome burnished ware	NC
5/2015		handmade red-black/monochrome burnished ware	NC
3593		handmade red-black/monochrome burnished ware	VCEm
3559		handmade red-black/monochrome burnished ware	VCEm
224/14		handmade red-black/monochrome burnished ware	VMgne
229/14		handmade red-black/monochrome burnished ware	VMgne
231/14		handmade red-black/monochrome burnished ware	VMgne
234/14		handmade red-black/monochrome burnished ware	VMgne
235/14		handmade red-black/monochrome burnished ware	VMgne
228/14		handmade red-black/monochrome burnished ware	NC
236/14		handmade red-black/monochrome burnished ware	VMmetag
1/2015		handmade red-black/monochrome burnished ware	VMmetag
3/2015		handmade red-black/monochrome burnished ware	VMmetag
253/14*		handmade kitchen ware	NC
254/14*		handmade kitchen ware	NC
250/14*		handmade kitchen ware	VMmetag
255/14*		handmade kitchen ware	VMmetag
249/14*		handmade kitchen ware	VMgne
252/14*		handmade kitchen ware	VMgne
247/14*		wheel-finished light-colored semifine ware	VCEm-a+Ib
3554		wheel-finished mass-produced bowl	VCEm-a
3555		wheel-finished light-colored semifine ware	VCEm-a
241/14*		wheel-finished light-colored fine ware	VCEm-a
240/14*		wheel-finished light-colored fine ware	VCIb
242/14*		wheel-finished light-colored fine ware	VCIb
3548		wheel-finished light-colored fine ware	CIb
3600		wheel-finished light-colored fine ware	CEb-m
248/14*		wheel-finished light-colored semifine ware	CEb-m
3551		wheel-finished light-colored fine ware	CEm-a
239/14*		wheel-finished light-colored fine ware	CEm-a
3550		wheel-finished light-colored fine ware	CIb
238/14*		wheel-finished light-colored fine ware	CIb
3601		wheel-finished light-colored fine ware	NC
COLL206/16*		wheel-finished mass-produced bowl	VC
COLL202/16*		wheel-finished mass-produced bowl	VCEm-a+Ib
COLL222/16*		wheel-finished mass-produced bowl	VCEm-a+Ib
COLL208/16*		wheel-finished mass-produced bowl	VC
COLL163/16*		wheel-finished light-colored semifine ware	VCEm-a+Ib
COLL219/16*		wheel-finished light-colored semifine ware	VCEm-a+Ib
COLL188/16*		wheel-finished light-colored fine ware	VCIb

Each petrographic group is mentioned according to the following acronyms: V = organic tempered pastes; C = calcareous clay; E, M and I = Inclusions of effusive, metamorphic and intrusive origin; b, m, a = basic, intermediate and acid composition; for the metamorphic rocks gne, metag and qu-sc are abbreviations of gneiss, metagabbro and quartz-schist. NC (not classifiable) refers to petro-loners. The samples marked with an asterisk are new, while the other ones have been already published [74, 84, 85].

**Table 3:** Main features of the petro-groups considered in this paper.

Petro-group	Main inclusions	Inclusion max. size/amount
**C**	ca, for, qu, bt	1.4mm/7%
**CEb-m**	ca, pl, qu, basaltic andesite, bt, qu-kfds aggregate, amph, cpx, ox, mu	0.7mm/15%
**CEm-a**	ca, trachyte-rhyolite, limestone, pl, ox, mu, amph, bt	1.4mm/7%
**CIb**	gabbro, pl, ca, for	2mm/10%
**VC**	veg, qu, ca, pl	3.3mm/7%
**VCEm**	veg, ca, pl, andesite, amph, bt, cpx, pumice, qu, ox	2mm/25%
**VCEm-a**	veg, ca, trachyte-rhyolite, limestone, qu, pl, ox, amph, bt, mu, sandstone	3.7mm/10%
**VCEm-a+Ib**	veg, ca, trachyte-rhyolite, gabbro, pl, qu, limestone, amph, ox, sandstone	5.6mm/10%
**VCIb**	veg, ca, gabbro, pl, ox, cpx, trachyte-rhyolite, limestone, sandstone, for, granite, qu, opx	7mm/15%
**VEm**	veg, pl, andesite, amph, bt, qu, cpx, pumice, opx	3.7mm/25%
**VIa**	veg, granite, qu, kfds, pl, bt, amph	2mm/15%
**VIb**	veg, gabbro, pl, ox, trachyte-rhyolite, qu, granite	7.8mm/20%
**VIm**	veg, diorite, qu, pl, kfds, amph	4mm/20%
**VMgne**	veg, gneiss, qu, amph, pl, bt, kfds	5mm/20%
**VMmetag**	veg, metagabbro, cpx, gneiss, amphibolite, qu, pl, kfds	5.17mm/24%
**VMqu-sc**	veg, mu-schist, qu-schist, mu, qu, bt, ox	4.8mm/30%

The types of inclusions are listed in decreasing order of importance. Abbreviations: veg = vegetal fibers; ca = calcite; pl = plagioclase; qu = quartz; bt = biotite; amph = amphibole; cpx = clinopyroxene; mu = muscovite; for = foraminifera; Kfds = K-feldspar; ox = oxide; opx = orthopyroxene. Further details have been reported in previous publications [74, 84, 85].

### Assessing the variability of metric data: Pottery elemental concentrations

The geochemical variability was quantified by calculating the coefficient of variation (CV) for each element concentration measured through wavelength-dispersive X-ray fluorescence, namely SiO_2_, TiO_2_, Fe_2_O_3_, MnO, MgO, CaO, Na_2_O, K_2_O, P_2_O_5_, V, Cr, Ni, Zn, Rb, Sr, Zr and Ba. The CV is defined as the ratio between standard deviation and mean, often multiplied by 100 to be expressed as a percentage. The higher the CV, the more variable the dataset. The CV has been commonly used not only in natural sciences, medicine and psychology but also in archaeological studies on vessel formal and dimensional standardization. As shown by the latter, it differs from other indexes in providing reliable measures of variability independently of sample size and the measure of scale [22, 88–90]. Blackman and colleagues [36] also successfully used the CV to assess the geochemical variability of the 3^rd^ millennium mass-produced bowls from Tell Leilan in northeast Syria.

Following a method proposed by Eerkens and Bettinger [22] for assessing the formal standardization of various archaeological artifacts, a scatter plot includes the mean and standard deviation of each element upon which the regression line is plotted. The regression line slopes vary according to the data variability: steeper slopes denote more variation in elemental concentrations. Furthermore, skewness and kurtosis were taken into account to estimate to what extent the data diverge from a normal distribution. In some studies on vessel formal standardization, these criteria have proven to be even more efficient than the CV to distinguish different levels of potters’ skills [90]. The skewness refers to the degree of distortion from a symmetrical data distribution, while the kurtosis measures the tailedness of this distribution, providing an indication of the presence of outliers. The closer to zero values the skewness and kurtosis are, the more normal is the distribution of data. Both skewness and kurtosis were calculated via the formulas available on Excel based on Fisher’s coefficient:

$$Skewness=\frac{n}{\left(n-1)(n-2\right)}\;\sum {\left(\frac{x_{i}-\overset{-}{x}}{s}\right)}^{3}$$

$$Kurtosis=\{\frac{n(n+1)}{\left(n-1)(n-2)(n-3\right)}\sum {\left(\frac{x_{i}-\overset{-}{x}}{s}\right)}^{4}\}-\frac{3{\left(n-1\right)}^{2}}{\left(n-2)(n-3\right)}$$

where n is the number of variables, x_i_ the i^th^ random variable, $\overset{-}{x}$ the mean of the distribution and s the standard deviation of the distribution.

The CVs calculated separately on each element have the disadvantage of overlooking the correlations between elemental patterns existing in ceramic artifacts. To obviate this, a series of variation matrixes (S1 Table) were produced following the method introduced by Aitchison [91, 92] and further developed for pottery analysis by Buxeda i Garrigós and Kilikoglou [37, 93]. Variation matrixes are defined by the variances of the natural log-ratios calculated on every pair of elements present in the data set. From the variation matrix one can calculate the total variation, which quantifies the variability of the data set and is also related to the Euclidean distances among all specimens [94]. The total variation is defined as the sum of all the variances in the variation matrix divided by two times the number of elements determined. The variation matrix can also be used to determine the variance of an element, which is equal to the sum of the variances calculated on all the log-ratios that use this element as divisor. This value gives an estimate of the contribution of this element to the total variation of the data set [91, 93]. In ceramic studies the total variation has frequently been applied to estimate intra-deposit variations, post-depositional alterations as well as the monogenic vs. polygenic nature of the data set. However, it is rarely coupled with thin section petrography to assess the level of standardization of raw material procurement and processing.

### Assessing the variability of non-metric data: Pottery petrographic grouping

**Fig 7:**
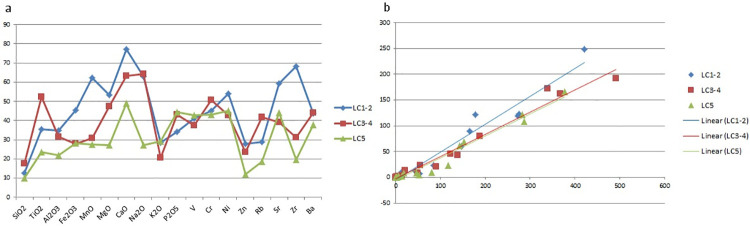
a. Mean of the CVs calculated for each element within each LC sub-phase; b. Relationships between mean (x-axis) and standard deviation (y-axis) for all chemical elements within each LC sub-phase.

Petrographic analyses of archaeological vessels usually aim at grouping thin sections into reference groups that ideally represent the ceramic pastes prepared in a certain way and place. The results are non-metric classifications similar to those obtained through typological methods. To assess the variability of such non-metric classification I applied three necessary and inextricably linked properties of diversity, which are employed across a full range of disciplines according to different degrees of prioritization and terminologies [95–97]. Here I will call these properties richness, evenness, and disparity (Fig 7). Richness can be also referred to as “variety”, and considers the number of categories—represented by petro-groups in this paper—in which elements are sorted. Evenness quantifies how equal is the distribution of elements across categories. In the present case it expresses how ceramic thin sections are distributed into each petro-group. Thus, evenness is analogous to statistical variance and can also be defined as “balance” or “concentration”. Ecological studies tend to focus on questions of richness and evenness due to the occurrence of well-established taxonomic schemes [96]. The concept of disparity—taken from paleontology and extensively used in conservation biology—indicates to what extent categories, for instance petro-groups, are different from each other, and is usually based on some form of distance measure. Typically, the greater the richness, evenness and disparity, the greater the diversity.

To quantify richness, evenness and variety I applied several indexes to the petrographic classification (Table 4). As for richness, I first considered the percentage of petro-loners. Petro-loners are composed of minerals and rocks of all local origin but differently combined with each other and in distinct grain-size distributions compared to the samples classified into petro-groups. In other words, these are vessels produced with different local deposits and/or recipes. Thus, petro-loners are random local recipes, which are comparable to *unica* in taxonomic classifications. Within single categories (e.g. periods, wares, manufacturing techniques) petro-groups that are represented by only one sample have been counted as petro-loners, even though they share features with samples outside the considered category. For instance, the handmade kitchen ware 262/14 is a loner within the handmade wares of the LC3-4, but not within the LC3-4 as a whole, since in this period two wheel-finished vessels (samples 261/14 and 299/14) present the same recipe (petro-group VEm). The richness was also quantified through the Menhinick’s and Shannon’s indexes (S2 Table), both commonly adopted in the ecological literature as a measure of biodiversity [98]. The Mehinick’s index is a simple species counting that attempts to reduce the effect of sample size on richness quantification, i.e. increased richness with larger sampling, by dividing the number of species recorded by the number of individuals in the sample. It is given here by the number of petro-groups divided the square root of the number of thin sections analyzed. The Shannon’s index was originally used within information theory to measure the entropy contained in a text based on the number and abundance of letter types [99]. The idea behind ecological applications is that the diversity of a community is similar to the amount of information in a code or message. For the purpose of calculations, the number of samples recurring in each recipe, including both petro-groups and loners, was divided by the total number of samples; this proportion was multiplied by its natural logarithm; the resulting product was summed across recipes and multiplied by -1:

$$Shannon’s\;index=-\sum_{i=1}^{s}p_{i}\;lnp_{i}$$

where p_i_ is the proportion of the population made of species i and s the number of species.

**Table 4:** Parameters considered for assessing the three different properties of diversity at a petrographic level.

**Richness**	% petro-loners
	Menhinick’s index
	Shannon’s index
**Evenness**	Highest disparity in recipe abundance
	Average number of samples per petro-group
	Pielou’s index
	Shannon’s index
**Disparity**	Jaccard’s dissimilarity %

Since Shannon’s index considers not only the number of petro-groups but also the distribution of thin sections into petro-groups, it has also been considered to assess the evenness. Evenness was also evaluated through the relative abundance of each recipe and especially through the maximum difference in abundance between the most and the least represented recipe. Both petro-groups and petro-loners were counted as more and less established recipes, respectively. In order to assess the evenness of only well-established recipes a further parameter was calculated by excluding the petro-loners, namely the average number of samples per petro-group. Last but not least, I calculated the Pielou’s index (S2 Table), which is obtained by dividing the Shannon’s index with the highest possible value this index could have in case of highest variability. Disparity measures are generally based on distances or dissimilarity coefficients, which indicate how dissimilar two cases are considering simultaneously all the variables for which they have been defined [100]. Dissimilarity coefficients are obtained by subtracting 1 from similarity coefficients. There are different similarity/dissimilarity coefficients according to the considered variables, of either a quantitative or qualitative nature. In this paper, I took into account and converted into percent the Jaccard distance based on the presence and absence of some basic ingredients that may occur across different petro-groups (S3 Table):

Jaccard’sdissimilarity%=1-Jaccard’scoefficient*100

where

$$Jaccard’s\;coefficient\;=\;\frac{number\;of\;present-present\;matches}{number\;of\;present-present\;matches+mismatches}$$

These basic ingredients correspond to the main discriminating criteria adopted for grouping ceramic thin sections [85] and are registered in the acronyms of each petro-group (Table 2). These are organic temper (V), calcareous matrix (C), granite (Ia), diorite (Im), quartz-schist (qu-sc), gabbro (Ib), trachyte-rhyolite (Em-a), andesite (Em), basaltic andesite (Eb-m), metagabbro (metag) and gneiss (gne). The Jaccard’s distance has not been calculated on petro-loners, which in a sense already represent an index of maximal disparity due to their lack of affinity with any other sample. While the assessment of disparity finds many applications in archaeology (e.g. cemetery analyses), richness and evenness are rarely considered even in specialized handbooks [100]. However, these latter indexes allow us to further nuance the concept of diversity and could be successfully applied to any kind of archaeological classification—e.g. morpho-functional, typological and stylistic—beyond standardization studies.

In summary, a high standardization of ceramic recipes should ideally correspond to low values of all diversity indexes (i.e. Menhinick’s, Shannon’s, Pielou’s and Jaccard’s), a reduced number of petro-loners, an unequal distribution of samples across petro-groups, and a high average number of samples per petro-groups.

## Results

### Geochemical homogenization as a result of production serialization

**Fig 8:**
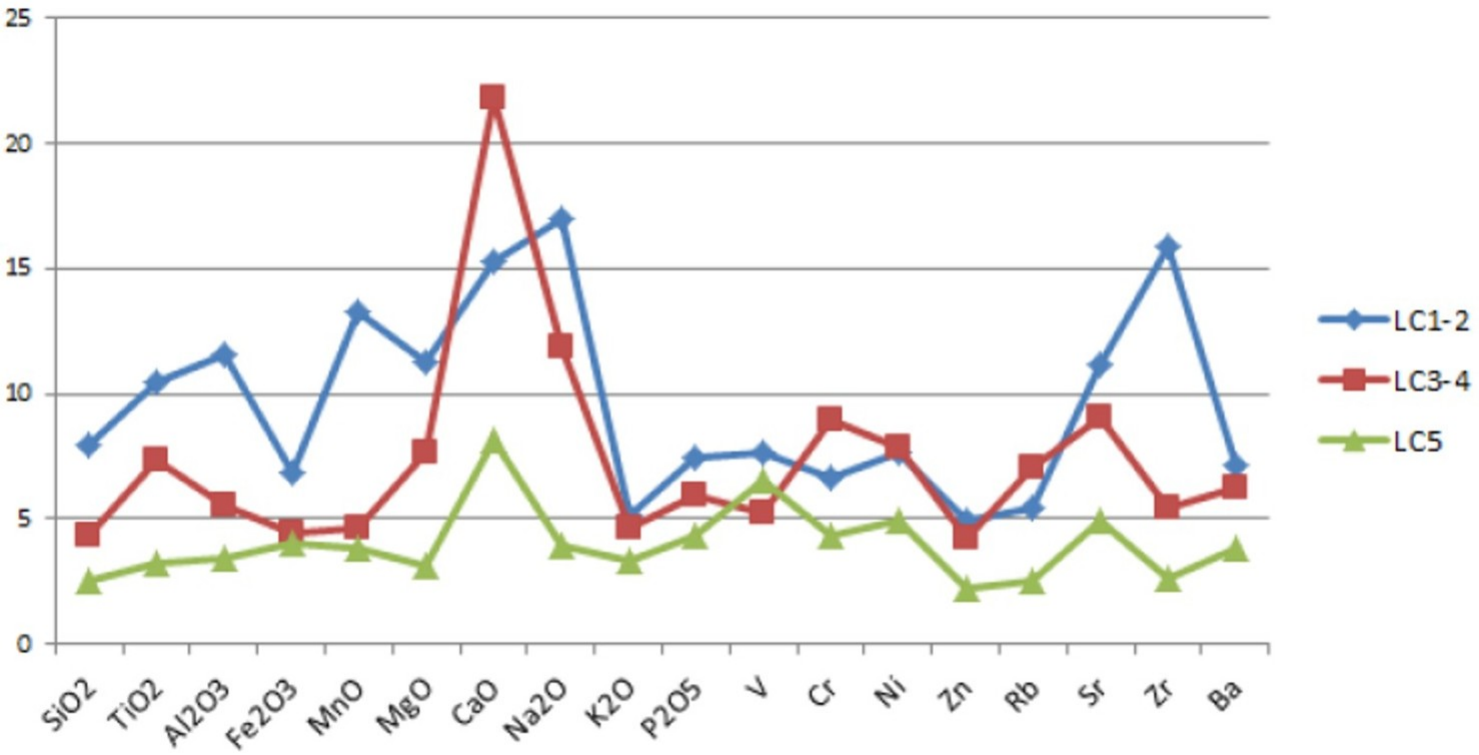
Elemental variance within each LC sub-phase. The variance of an element is equal to the trace of the variance-covariance matrix of the log-ratio transformed data using this element as divisor [91, 93].

In order to compare each Late Chalcolithic phase—i.e. LC1-2 (Arslantepe VIII), LC3-4 (VII) and LC5 (VI A)—I plotted on a line graph the mean of the CVs calculated for each element (Table 5 and Fig 7a) and I found that the geochemical variability tends to decrease throughout the LC period in terms of both major and trace elements. An identical trend can be inferred from the scatterplot (Fig 7b) relating the standard deviation with the mean of all elements: the regression line of the LC1-2 is steeper compared to those of the following phases, suggesting a higher compositional variability. The geochemical homogenization across the Late Chalcolithic becomes even more pronounced when considering the elemental variance and the total variation (Fig 8, Table 5). The elements responsible for the highest variability of the first Late Chalcolithic phase are Al_2_O_3_, TiO_2_, MnO, MgO, Na_2_O and Zr.

**Table 5:** Major (weight %) and trace element (parts per million) concentrations as well as associated means, standard deviations (dev std), coefficients of variation (CV), skewness, kurtosis, elemental variance and total variation within each LC sub-phase.

Sample	Phase	Ceramic ware	SiO_2_	TiO_2_	Al_2_O_3_	Fe_2_O_3_	MnO	MgO	CaO	Na_2_O	K_2_O	P_2_O_5_	V	Cr	Ni	Zn	Rb	Sr	Zr	Ba
134/14	**LC1-2**	handmade burnished ware	51.03	0.70	12.39	7.48	0.11	6.64	17.31	1.16	3.00	0.18	147	531	323	103	59	487	103	226
201/15		handmade mass-produced bowls	45.49	0.59	10.90	5.21	0.07	5.38	28.39	1.04	2.67	0.26	110	287	223	92	40	814	121	248
208/15		handmade mass-produced bowls	48.42	1.24	13.17	7.13	0.09	4.11	21.20	1.47	2.91	0.25	130	291	177	104	43	457	124	465
210/15		handmade burnished ware	63.94	1.32	27.37	1.98	0.01	0.41	3.64	0.11	1.08	0.15	106	161	63	37	16	70	473	65
211/15		handmade burnished ware	60.49	0.81	20.43	5.76	0.04	1.91	4.95	3.28	2.17	0.16	91	147	79	67	55	484	163	307
212/15		handmade plain ware	56.09	1.42	18.42	12.19	0.18	2.91	4.74	1.89	1.92	0.23	263	305	156	107	46	201	166	210
215/15		handmade mass-produced bowls	46.99	0.60	11.08	5.29	0.08	5.04	26.49	1.02	3.14	0.29	110	298	218	80	46	646	127	379
221/15		handmade plain ware	58.62	0.87	18.22	9.70	0.17	3.67	4.03	2.35	2.28	0.09	211	177	83	88	45	204	147	279
**mean**			**53.88**	**0.94**	**16.50**	**6.84**	**0.09**	**3.76**	**13.84**	**1.54**	**2.39**	**0.20**	**146.14**	**274.76**	**165.20**	**84.80**	**43.75**	**420.48**	**177.86**	**272.34**
**dev std**			**6.85**	**0.34**	**5.72**	**3.10**	**0.06**	**2.00**	**10.69**	**0.97**	**0.69**	**0.07**	**60.27**	**123.42**	**89.16**	**23.60**	**12.68**	**248.83**	**121.16**	**119.19**
**CV**			**12.71**	**35.51**	**34.67**	**45.28**	**62.19**	**53.29**	**77.22**	**62.78**	**28.67**	**33.99**	**41.24**	**44.92**	**53.97**	**27.83**	**28.99**	**59.18**	**68.12**	**43.77**
**skewness**			**0.19**	**0.43**	**0.93**	**0.33**	**0.27**	**-0.34**	**0.32**	**0.55**	**-0.96**	**-0.30**	**1.35**	**1.26**	**0.54**	**-1.28**	**-1.48**	**0.11**	**2.65**	**-0.10**
**kurtosis**			**-1.65**	**-1.79**	**0.39**	**0.53**	**-0.64**	**-0.27**	**-2.10**	**0.61**	**0.66**	**-1.07**	**0.86**	**2.28**	**-0.33**	**1.46**	**3.53**	**-0.77**	**7.23**	**0.85**
**variance**			**7.94**	**10.43**	**11.61**	**6.85**	**13.27**	**11.22**	**15.29**	**16.96**	**5.18**	**7.41**	**7.65**	**6.66**	**7.67**	**4.96**	**5.41**	**11.15**	**15.89**	**7.15**
**total variation**			**4.80**																	
301/14	**LC3-4**	wheel-finished mass-produced bowl	44.41	0.44	9.18	3.95	0.07	4.03	33.25	0.91	3.37	0.39	81	306	154	69	53	746	111	263
304/14		wheel-finished mass-produced bowl	37.46	0.53	8.89	4.58	0.08	3.55	41.24	0.42	3.00	0.26	77	394	146	79	32	478	117	236
109/14		wheel-finished mass-produced bowl	45.75	0.56	10.72	5.02	0.08	4.57	29.68	0.75	2.64	0.23	92	515	182	67	55	542	130	263
285/14		wheel-finished mass-produced bowl	39.16	0.41	8.82	4.76	0.06	4.25	38.02	0.67	3.57	0.27	76	315	238	106	29	884	96	375
302/14		wheel-finished mass-produced bowl	48.41	0.50	11.43	5.10	0.08	4.56	25.36	0.89	3.37	0.30	74	223	211	93	56	475	115	275
303/14		wheel-finished mass-produced bowl	38.48	0.50	8.70	4.84	0.07	5.71	38.78	0.35	2.39	0.17	91	604	246	83	39	541	109	195
192/15		wheel-finished mass-produced bowl	49.35	0.50	11.27	4.53	0.08	4.46	25.27	1.16	3.06	0.31	70	329	162	60	64	549	127	331
193/15		wheel-finished mass-produced bowl	33.55	0.30	6.36	4.45	0.07	10.54	43.00	0.23	1.37	0.12	71	634	257	69	21	558	53	399
171/15		wheel-finished mass-produced bowl	33.94	0.35	6.56	4.89	0.08	10.28	42.17	0.29	1.22	0.21	90	786	286	67	23	682	65	118
271/14		wheel-finished red-slipped burnished ware	62.29	0.96	19.03	8.93	0.15	2.62	0.89	1.22	3.70	0.22	160	307	147	137	104	109	189	685
300/14		wheel-finished red-slipped burnished ware	37.01	0.64	8.46	5.02	0.08	8.27	37.70	0.76	1.92	0.14	116	329	244	76	37	770	101	218
103/14		wheel-finished red-slipped burnished ware	48.28	1.69	13.42	7.64	0.10	5.25	18.87	1.95	2.52	0.29	201	226	171	100	50	636	164	457
157/14		wheel-finished red-slipped burnished ware	44.95	0.92	11.63	6.25	0.08	6.25	25.36	1.53	2.79	0.22	100	326	239	93	31	518	108	381
160/14		wheel-finished red-slipped burnished ware	43.46	1.16	10.76	6.39	0.08	9.25	24.85	1.40	2.47	0.19	152	549	232	102	29	568	133	284
161/14		wheel-finished red-slipped burnished ware	47.26	0.54	10.49	5.02	0.08	5.72	26.73	0.91	3.05	0.18	90	545	243	88	57	533	121	293
162/14		wheel-finished red-slipped burnished ware	42.47	0.51	9.31	5.37	0.09	6.38	32.19	0.55	2.93	0.20	98	526	301	95	39	753	109	239
163/14		wheel-finished red-slipped burnished ware	45.12	0.42	9.05	4.58	0.07	6.96	29.79	0.98	2.86	0.16	79	588	274	97	47	669	97	260
308/14		wheel-finished red-slipped burnished ware	48.33	1.28	12.98	6.62	0.08	3.64	21.56	1.42	3.39	0.70	154	392	191	84	48	473	155	237
310/14		wheel-finished red-slipped burnished ware	40.97	0.40	8.59	4.83	0.08	8.41	33.02	0.73	2.81	0.15	71	360	298	93	28	677	83	354
282/14		wheel-finished light-colored fine ware	40.84	0.43	8.79	6.00	0.09	7.76	32.20	0.86	2.63	0.41	103	427	358	99	25	802	95	404
284/14		wheel-finished light-colored fine ware	43.68	0.53	9.23	5.03	0.10	4.99	32.63	0.53	3.00	0.28	90	592	230	88	43	608	117	247
286/14		wheel-finished light-colored fine ware	46.75	0.63	10.65	6.33	0.13	5.84	25.92	0.45	2.98	0.31	126	332	287	106	44	535	111	715
287/14		wheel-finished light-colored fine ware	51.34	1.10	14.71	7.22	0.09	4.60	15.88	1.81	3.06	0.19	141	222	161	111	45	335	112	462
288/14		wheel-finished light-colored fine ware	54.83	0.99	16.80	10.26	0.14	7.33	5.75	1.35	2.34	0.20	168	345	308	108	63	115	93	457
105/14		wheel-finished chaff-tempered ware	48.98	1.52	13.63	7.40	0.10	5.11	18.55	1.64	2.79	0.27	167	249	194	89	45	598	164	312
257/14		wheel-finished chaff-tempered ware	61.63	1.22	18.18	6.91	0.09	2.46	3.76	2.72	2.83	0.20	122	156	87	72	74	329	194	383
106/14		wheel-finished chaff-tempered ware	54.89	0.92	14.00	5.89	0.08	3.40	15.31	1.85	3.36	0.31	113	266	154	78	74	510	147	308
258/14		wheel-finished chaff-tempered ware	49.21	1.74	14.68	7.48	0.10	3.72	18.05	2.20	2.59	0.24	180	197	126	87	48	597	185	295
260/14		wheel-finished chaff-tempered ware	51.66	1.68	14.46	7.68	0.11	4.56	14.29	2.23	2.88	0.45	201	300	187	99	54	544	167	275
281/14		wheel-finished chaff-tempered ware	62.09	1.26	18.07	6.69	0.09	2.57	3.87	2.89	2.31	0.17	119	133	72	70	78	347	217	413
272/14		handmade light-colored ware	52.33	0.82	13.03	6.17	0.07	3.15	20.21	1.01	2.95	0.26	136	314	140	91	58	356	167	377
273/14		handmade light-colored ware	53.25	1.76	15.51	7.98	0.08	2.98	13.48	2.22	2.54	0.20	183	204	98	96	61	392	196	264
277/14		handmade kitchen ware	55.58	1.56	17.57	12.66	0.20	3.68	4.25	1.90	2.22	0.37	265	191	112	160	42	223	161	433
262/14		handmade kitchen ware	60.48	0.80	20.47	5.07	0.09	2.27	5.02	3.52	2.15	0.13	85	142	63	58	89	477	175	316
278/14		handmade kitchen ware	48.97	0.65	11.22	7.45	0.14	4.11	23.80	0.45	3.00	0.20	134	643	291	106	51	435	95	661
280/14		handmade kitchen ware	64.37	1.02	18.28	6.45	0.05	2.98	1.25	1.18	4.18	0.23	144	223	167	130	128	205	225	514
307/14		wheel-finished kitchen ware	63.16	0.56	15.65	6.62	0.10	3.19	5.74	1.42	3.43	0.14	152	235	116	69	84	169	141	326
259/14		wheel-finished kitchen ware	56.90	2.02	18.40	8.73	0.11	2.67	5.35	3.26	2.31	0.25	182	118	66	83	63	401	231	308
261/14		wheel-finished kitchen ware	59.84	0.84	20.92	5.71	0.09	1.97	4.82	3.05	2.29	0.49	93	127	83	68	70	476	179	417
274/14		handmade red-black/monochrome burnished ware	63.02	0.91	17.01	8.06	0.08	1.92	4.70	0.73	3.23	0.32	138	167	98	80	80	90	158	1002
291/14		handmade red-black/monochrome burnished ware	62.91	0.66	20.19	4.43	0.05	1.70	3.86	3.15	2.69	0.36	60	79	53	65	84	460	159	301
**mean**			**49.69**	**0.88**	**13.10**	**6.32**	**0.09**	**4.82**	**20.64**	**1.40**	**2.78**	**0.26**	**122.93**	**339.38**	**187.10**	**89.42**	**54.75**	**491.76**	**138.27**	**367.17**
**dev std**			**8.71**	**0.46**	**4.11**	**1.77**	**0.03**	**2.28**	**13.06**	**0.90**	**0.57**	**0.11**	**45.85**	**172.50**	**79.71**	**20.98**	**22.87**	**191.51**	**43.03**	**161.84**
**CV**			**17.53**	**52.42**	**31.39**	**28.00**	**30.70**	**47.34**	**63.26**	**64.10**	**20.60**	**43.08**	**37.29**	**50.83**	**42.60**	**23.47**	**41.78**	**38.94**	**31.12**	**44.08**
**skewness**			**0.08**	**0.85**	**0.31**	**1.42**	**2.06**	**0.91**	**0.01**	**0.83**	**-0.42**	**1.82**	**0.91**	**0.72**	**0.11**	**1.15**	**0.99**	**-0.35**	**0.31**	**1.99**
**kurtosis**			**-0.89**	**-0.33**	**-1.07**	**2.95**	**5.85**	**0.21**	**-1.24**	**-0.24**	**1.23**	**5.02**	**0.73**	**-0.25**	**-0.92**	**2.19**	**1.40**	**-0.11**	**-0.51**	**5.34**
**variance**			**4.36**	**7.35**	**5.51**	**4.47**	**4.64**	**7.61**	**21.76**	**11.82**	**4.58**	**5.89**	**5.23**	**8.99**	**7.85**	**4.27**	**7.00**	**9.02**	**5.43**	**6.26**
**total variation**			**3.67**																	
239/14	**LC5**	wheel-finished light-colored fine ware	49.76	0.63	11.88	5.15	0.09	4.36	23.36	1.18	3.42	0.16	98	250	202	70	58	532	120	340
241/14		wheel-finished light-colored fine ware	44.62	0.40	9.08	4.41	0.07	7.00	30.21	1.04	3.01	0.16	50	542	260	91	33	554	87	514
242/14		wheel-finished light-colored fine ware	49.18	0.71	11.64	5.62	0.09	5.00	22.47	1.20	3.84	0.26	101	315	200	91	59	508	123	403
247/14		wheel-finished light-colored semifine ware	45.85	0.60	10.55	5.10	0.09	5.52	28.20	1.32	2.49	0.28	107	413	247	77	50	640	109	278
248/14		wheel-finished light-colored semifine ware	52.46	0.70	11.09	5.81	0.10	3.55	22.20	1.05	2.60	0.42	111	379	181	90	45	439	130	349
249/14		handmade kitchen ware	58.96	0.71	15.30	7.47	0.12	3.11	10.18	1.89	2.15	0.11	168	131	72	68	39	222	113	199
250/14		handmade kitchen ware	58.42	0.78	17.77	9.43	0.15	4.55	4.73	1.99	2.06	0.13	221	251	104	77	54	172	114	222
252/14		handmade kitchen ware	57.92	0.73	14.70	7.46	0.12	3.28	11.84	1.62	2.23	0.11	149	167	81	71	39	193	115	200
253/14		handmade kitchen ware	46.04	0.87	13.44	8.87	0.12	4.22	22.80	1.27	2.10	0.26	178	238	114	94	37	279	134	162
254/14		handmade kitchen ware	51.83	1.09	15.61	8.03	0.14	3.46	15.84	1.93	1.82	0.26	125	246	146	77	46	376	166	287
255/14		handmade kitchen ware	55.34	0.74	17.89	10.40	0.17	6.07	5.72	2.12	1.37	0.19	263	172	76	80	43	232	80	191
**mean**			**51.85**	**0.72**	**13.54**	**7.07**	**0.11**	**4.56**	**17.96**	**1.51**	**2,46**	**0.21**	**142.80**	**282.18**	**152.92**	**80.55**	**45.76**	**376.91**	**117.25**	**285.91**
**dev std**			**5.26**	**0.17**	**2.95**	**1.98**	**0.03**	**1.24**	**8.79**	**0.41**	**0,72**	**0.09**	**61.23**	**121.89**	**68.73**	**9.65**	**8.54**	**166.36**	**22.91**	**107.78**
**CV**			**10.14**	**23.43**	**21.75**	**28.08**	**27.41**	**27.26**	**48.97**	**27.09**	**29,28**	**44.46**	**42.88**	**43.20**	**44.94**	**11.98**	**18.66**	**44.14**	**19.54**	**37.70**
**skewness**			**0.07**	**0.40**	**0.15**	**0.29**	**0.56**	**0.73**	**-0.27**	**0.32**	**0,63**	**0.95**	**0.66**	**0.96**	**0.29**	**0.20**	**0.27**	**0.20**	**0.39**	**0.93**
**kurtosis**			**-1.49**	**2.43**	**-1.14**	**-1.21**	**-0.53**	**-0.24**	**-1.29**	**-1.77**	**0,05**	**0.93**	**0.19**	**0.65**	**-1.44**	**-1.56**	**-1.07**	**-1.60**	**1.39**	**0.41**
**variance**			**2.57**	**3.20**	**3.42**	**4.03**	**3.85**	**3.15**	**8.12**	**3.95**	**3,28**	**4.31**	**6.58**	**4.33**	**4.96**	**2.18**	**2.49**	**4.93**	**2.60**	**3.80**
**total variation**			**1.99**																	

**Fig 9:**
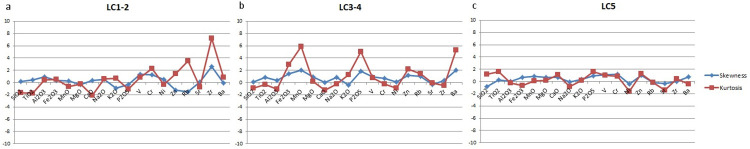
Skewness and kurtosis calculated for each element within the LC1-2 (a), LC3-4 (b) and LC5 (c).

The diachronic trend towards normality revealed by the skewness and kurtosis (Table 5 and Fig 9a–9c) is not as gradual as that towards homogeneity mentioned above: after the LC1-2 (Fig 9a), the LC3-4 marks a break distinguished by the most asymmetric and heavy-tailed distribution of data due especially to Fe_2_O_3_, MnO, P_2_O_5_, Zn and Ba concentrations (Fig 9b), followed by the final Late Chalcolithic phase (5) that shows the highest normality (Fig 9c).

**Fig 10:**
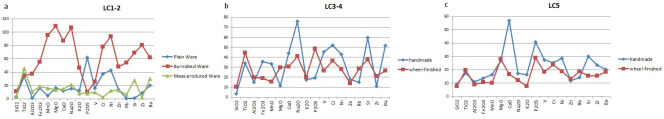
Relationships between elemental CVs and ceramic wares/manufacturing techniques found in the LC1-2 (a), LC3-4 (b) and LC5(c).

**Fig 11:**
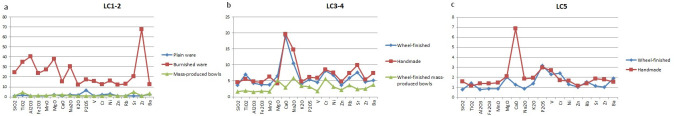
Relationships between elemental variances and ceramic wares/manufacturing techniques found in the LC1-2 (a), LC3-4 (b) and LC5(c).

Within each Late Chalcolithic sub-phase, the variability indexes noticeably fluctuate according to the production rate and manufacturing techniques (Tables 6 and 7; Figs 10a–10c and 11a–11c). In the first Late Chalcolithic phase, when the whole production is still entirely handmade, the mass-produced bowls show slightly lower values of elemental CVs and variances as well as of total variation (Tables 6 and 7; Figs 10a and 11a), while the burnished ware exhibits the highest geochemical variability for all the considered parameters. In the following phases (Tables 6 and 7; Figs 10b, 10c, 11b and 11c), that part of the assemblage which is now shaped on the wheel is chemically more homogeneous than handmade vessels. The calculations on LC3-4 wheel-finished vessels also include mass-produced bowls; when extrapolated, mass-produced bowls show a wider gap with the rest of the wheel-finished vessels (difference in total variation = 1.67) than that separating these latter from handmade exemplars (difference in total variation = 0.5). Chemical CVs and total variations calculated separately (S1 Table; Tables 8 and 9) on each single ware of the LC3-4 period evidence further interesting trends. The handmade monochrome/red-burnished and kitchen wares stand out for their chemical variability, while a much more homogeneous composition occurs in the wheel-finished mass-produced bowls and chaff-tempered smoothed ware as well as in the handmade light-colored ware. Intermediate values were instead obtained for the wheel-finished red-slipped burnished, kitchen and light-colored fine wares. Thus, the LC3-4 chemical variability is affected not only by the forming techniques and production rates but also by the type of surface treatments, firing conditions and the calcareous content of the clay matrix. Chemically more heterogeneous are the vessels with a non-calcareous clay matrix, burnished and fired in reducing or mixed atmospheres, such as the monochrome/red-burnished and kitchen wares. By contrast, more homogeneous compositions occur in calcareous-rich, light-colored, smoothed or plain vessels including the mass-produced, chaff-tempered smoothed and light-colored wares. In contrast, functionality does not play a significant role on the chemical standardization, as the same vessel shape might show very different chemical indexes. As opposed to LC3-4, the few wares of the LC5 period do not differ that much from each other in terms of chemical variability.

**Table 6:** Average CVs calculated on each element according to the different ceramic classes, manufacturing techniques and production rates occurring in the LC1-2, LC3-4 and LC5 phases.

Average CVs	LC1-2			LC3-4		LC5	
	Plain ware	Burnished ware	Mass-produced bowls	Handmade wares	Wheel-finished wares	Handmade wares	Wheel-finished wares
**SiO**_**2**_	3.12	11.43	3.13	3.27	10.37	9.17	7.69
**TiO**_**2**_	33.81	35.10	46.05	34.20	44.49	17.61	19.61
**Al**_**2**_**O**_**3**_	0.79	37.36	10.80	15.01	19.98	11.09	8.93
**Fe**_**2**_**O**_**3**_	16.10	55.48	18.53	35.58	18.80	13.63	10.61
**MnO**	4.28	95.25	15.98	33.35	15.37	16.31	10.06
**MgO**	16.39	108.92	13.63	11.53	29.86	26.92	27.95
**CaO**	11.58	87.33	14.70	43.92	30.72	56.85	16.77
**Na**_**2**_**O**	15.57	106.73	21.45	75.97	41.04	17.10	12.06
**K**_**2**_**O**	12.15	46.44	8.09	17.39	20.31	16.34	7.66
**P**_**2**_**O**_**5**_	62.24	9.95	7.91	19.23	48.52	40.96	29.03
**V**	15.65	25.28	9.96	45.32	26.45	27.44	18.41
**Cr**	37.69	77.93	1.92	52.00	36.48	25.22	23.81
**Ni**	42.80	93.89	12.20	43.06	28.23	28.55	18.62
**Zn**	14.04	47.97	13.17	17.89	13.69	11.96	13.03
**Rb**	0.62	54.24	6.61	15.02	28.51	14.39	18.69
**Sr**	1.01	69.16	27.94	59.78	37.92	30.01	15.34
**Zr**	8.80	80.78	2.42	11.19	21.04	23.56	15.36
**Ba**	19.96	61.90	30.02	51.87	26.74	20.21	18.47
**Mean**	**17.59**	**61.40**	**14.70**	**32.53**	**27.70**	**22.63**	**16.23**

**Table 7:** Elemental variance and total variation according to ceramic classes, manufacturing rates and production rates.

Phase	Ceramic classes	Total variation	Elemental variance																	
			SiO_2_	TiO_2_	Al_2_O_3_	Fe_2_O_3_	MnO	MgO	CaO	Na_2_O	K_2_O	P_2_O_5_	V	Cr	Ni	Zn	Rb	Sr	Zr	Ba
**LC1-2**	**Plain ware**	0.92	1.25	1.96	1.08	0.98	0.98	2.22	0.92	2.14	1.84	6.64	0.97	2.35	2.97	0.94	1.09	1.15	0.92	2.59
	**Mass-produced bowls**	0.88	0.88	4.69	0.95	1.34	1.32	1.98	2.20	1.46	0.88	1.09	0.93	0.93	1.81	0.89	0.88	4.89	0.91	3.48
	**Burnished ware**	12.08	24.64	34.74	40.44	23.86	27.28	37.97	15.28	30.52	12.07	17.30	15.63	12.52	16.18	12.07	13.00	20.74	67.69	12.81
**LC3-4**	**Wheel-finished**	3.19	3.72	7.11	4.26	3.88	3.79	6.53	18.85	10.58	4.09	5.45	4.56	8.15	6.78	3.68	5.89	7.72	4.73	5.22
	**Mass-produced bowls**	1.52	1.65	1.95	1.58	1.77	1.75	5.20	2.99	5.93	3.47	3.15	1.87	5.66	3.15	2.18	3.71	2.42	2.59	3.82
	**Handmade**	3.68	4.53	5.57	4.76	4.61	6.27	4.22	19.59	14.84	4.87	6.15	5.90	8.57	7.53	4.83	7.37	9.95	5.41	7.40
**LC5**	**Wheel-finished**	0.73	0.80	1.44	0.80	0.85	0.87	2.01	1.26	0.87	1.37	3.19	2.31	2.43	1.32	1.00	1.53	1.14	1.04	1.91
	**Handmade**	1.01	1.57	1.14	1.40	1.37	1.47	2.08	6.89	1.87	1.95	2.96	2.68	1.70	1.66	1.16	1.37	1.86	1.76	1.56

**Table 8:** Average CVs calculated on each element according to the different ceramic wares occurring in the LC3-4 and LC5 phases.

Average CVs	LC3-4 ceramic wares								LC5 ceramic wares		
	Handmade			Wheel-finished					Handmade	Wheel-Finished	
	Light-colored	Red-black/ monochrome burnished	Kitchen	Kitchen	Fine light-colored	Red-slipped burnished	Chaff-tempered smoothed	Mass-produced	Kitchen	Light-colored semifine	Light-colored fine
**SiO**_**2**_	1.24	0.12	11.59	5.22	11.91	14.56	10.80	14.53	9.17	9.50	5.88
**TiO**_**2**_	51.42	23.06	39.24	67.90	39.80	49.99	22.54	18.85	17.61	11.53	27.70
**Al**_**2**_**O**_**3**_	12.32	12.09	23.54	14.39	29.43	28.18	13.30	20.18	11.09	3.58	14.28
**Fe**_**2**_**O**_**3**_	18.07	41.16	41.91	22.07	28.70	23.02	9.42	7.50	13.63	9.19	12.03
**MnO**	10.16	33.89	55.47	13.08	24.42	24.41	9.73	7.49	16.31	10.74	9.38
**MgO**	3.96	8.68	24.81	23.51	22.91	33.37	29.18	46.67	26.92	30.69	25.20
**CaO**	28.25	13.83	119.75	8.68	51.35	40.59	55.02	20.00	56.85	16.83	16.72
**Na**_**2**_**O**	53.03	88.22	74.42	39.07	57.51	37.80	21.38	51.41	17.10	16.38	7.75
**K**_**2**_**O**	10.68	13.09	32.72	24.42	10.98	17.38	12.45	32.23	16.34	3.16	12.16
**P**_**2**_**O**_**5**_	18.80	7.70	42.73	61.25	31.91	67.75	36.90	32.06	40.96	28.78	29.29
**V**	20.94	55.71	48.93	31.95	24.54	34.77	24.74	10.77	27.44	2.27	34.55
**Cr**	29.86	50.51	77.12	40.65	35.82	30.42	30.17	41.16	25.22	6.07	41.56
**Ni**	24.82	42.63	62.16	28.92	28.18	21.96	37.25	24.14	28.55	21.85	15.40
**Zn**	3.70	15.06	37.74	11.74	9.09	16.78	13.50	19.30	11.96	11.24	14.82
**Rb**	3.09	3.11	50.76	14.94	30.55	47.44	24.01	39.17	14.39	8.07	29.30
**Sr**	6.90	95.10	42.03	45.92	54.97	33.30	24.78	22.60	30.01	26.35	4.33
**Zr**	11.36	0.27	32.86	24.60	10.36	26.68	13.93	26.05	23.56	12.61	18.11
**Ba**	25.09	76.12	30.15	16.69	36.85	41.78	16.40	32.05	20.21	15.90	21.04
**Mean**	**18.54**	**32.24**	**47.11**	**27.50**	**29.96**	**32.79**	**22.53**	**25.90**	**22.63**	**13.60**	**18.86**

**Table 9:** Elemental variance and total variation according to the different ceramic wares occurring in the LC3-4 and LC5 phases.

Phase	Ceramic wares		Total variation	Elemental variance																	
				SiO_2_	TiO_2_	Al_2_O_3_	Fe_2_O_3_	MnO	MgO	CaO	Na_2_O	K_2_O	P_2_O_5_	V	Cr	Ni	Zn	Rb	Sr	Zr	Ba
**LC3-4**	**Wheel-finished**	**Kitchen**	1.94	2.22	8.01	2.22	2.48	2.19	3.31	2.25	4.79	3.50	8.66	3.94	5.49	4.26	2.00	2.81	6.49	2.47	2.60
		**Mass-produced bowls**	1.52	1.65	1.95	1.58	1.77	1.75	5.20	2.99	5.93	3.47	3.15	1.87	5.66	3.15	2.18	3.71	2.42	2.59	3.82
		**Fine light-colored**	2.56	2.80	5.40	3.98	3.71	3.48	3.40	12.37	8.90	2.85	4.32	3.50	5.18	4.16	2.65	4.58	13.20	2.84	4.70
		**Red-slipped burnished**	3.01	3.37	6.25	3.94	3.63	3.88	6.20	24.84	4.93	3.70	5.96	4.48	5.20	4.52	3.56	5.87	8.91	3.85	5.33
		**Chaff-tempered smoothed**	1.26	2.07	1.98	2.23	1.45	1.44	1.96	9.25	2.92	1.58	2.45	1.74	2.19	2.99	1.28	3.38	1.82	2.19	2.54
	**Handmade**	**Light-colored**	1.06	1.06	5.71	1.21	1.47	1.15	1.15	2.86	6.06	1.40	1.93	1.64	3.06	2.48	1.06	1.06	1.08	1.18	2.51
		**Red-black/monochrome burnished**	4.26	4.38	4.66	5.01	6.35	5.50	4.26	4.31	26.73	4.30	4.72	8.86	7.82	6.55	4.34	4.49	31.77	4.39	14.86
		**Kitchen**	4.97	5.85	7.19	7.26	6.45	9.83	5.36	29.35	21.13	6.94	6.94	7.50	11.31	10.96	7.11	11.72	9.35	8.52	6.05
**LC5**	**Wheel-finished**	**Light-colored semifine**	0.47	0.65	0.73	0.50	0.64	0.69	2.17	0.95	0.93	0.49	2.05	0.48	0.52	1.30	0.71	0.57	1.70	0.78	0.95
		**Light-colored fine**	0.81	0.86	1.99	1.07	0.91	0.90	2.18	1.61	0.86	0.90	1.66	3.12	4.10	1.50	1.22	2.32	1.00	1.24	1.80
	**Handmade**	**Kitchen**	1.01	1.57	1.14	1.41	1.35	1.47	2.11	6.89	1.87	1.95	2.96	2.68	1.70	1.66	1.16	1.37	1.86	1.74	1.56

Independently of periods and wares, elemental CVs and variances are respectively higher for CaO, Na_2_O, Cr, V, Ni, P_2_O_5_, Sr, Ba and CaO, Na_2_O, Sr (Table 5; Figs 7 and 8). Based on the skewness and kurtosis the V, Cr, Zn and Rb concentrations diverge most extensively from a normal distribution (Table 5; Fig 9a–9c). Although some of these more variable elements are known to be sensitive to post-depositional processes (e.g. CaO, P_2_O_5_), most of them are instead related to distinct local strategies in raw material procurement and paste preparation. Indeed, previous studies have already demonstrated that the geochemical variation in the ceramics from Arslantepe is mostly linked to the exploitation of more and less calcareous clay deposits tempered with materials characterized by different mafic/felsic/alkaline affinities [85]. Calcareous and non-calcareous deposits are respectively available in the plain and in the southern Anti-Taurus Mountains. Clay pastes tempered with acid rocks (e.g. petro-groups CEm-a and VCEm-a) are richer in Ba, Rb, K_2_O, SiO_2_ and poorer in TiO_2_, Fe_2_O_3_, V, MnO, MgO, Cr and Ni. Opposite geochemical trends characterize the samples containing minerals and rocks of mafic origin (petro-groups CEb-m, CIb, VCIb, VIb, VMetag). In particular, metagabbroic pastes (petro-group VMetag) are strongly enriched in V, related to ultramafic rocks of ophiolite-related petrogenesis. Ceramic pastes with intermediate rocks (e.g. VCEm, VEm) show intermediate features between the terms mentioned above, but they are distinguished by high Al_2_O_3_, K_2_O, Na_2_O and Sr values.

### Petro-chemical discrepancies in diachronic trends towards standardization

The various indexes and forms applied to explore the petrographic variability of Late Chalcolithic vessel from Arslantepe (Table 10) evidence different trends than those obtained through the elaboration of geochemical data: at a petrographic level it is the LC3-4 and not the final LC5 that shows the lowest variability. Indeed, the lowest richness, evenness and disparity unequivocally characterize the LC3-4 phase, as the various diversity indexes provide the lowest values; petro-loners occur more rarely; samples are unevenly apportioned into petro-groups; and the average number of samples per petro-group is higher.

**Table 10:** Values of the diversity parameters considered for each LC sub-phase.

	RICHNESS		EVENNESS			RICHNESS+EVENNESS	DISPARITY
	% petro-loners	Menhinick’s	Disparity in recipe abundance	Average nr of samples per petro-group	Pielou’s	Shannon’s	Jaccard’s dissimilarity %
**LC1-2**	57.89	3.21	16	2.67	0.95	2.51	88.89
**LC3-4**	15.46	2.44	18	9.11	0.79	2.51	66.38
**LC5**	31.37	3.64	12	3.5	0.92	3	66.48

By applying the same parameters to the different wares within each Late Chalcolithic sub-phase it was possible to identify differences related to manufacturing techniques, ceramic style and traditions as well as production rates and morpho-functional features (Tables 9 and 10). Concerning the first Late Chalcolithic phase (LC1-2), the burnished ware is distinguished by the highest variability in terms of both richness and evenness (Table 11). The plain grit ware presents the highest petrographic homogeneity, closely followed by the mass-produced bowls and plain ware. Geochemical data are not available for the plain grit ware; however, they also evidenced a higher homogeneity for the mass-produced bowls. During the following LC3-4 period, the lowest petrographic variability occurs in the wheel-finished vessels. Diversity indexes provide lower values, petro-loners are rare, petro-groups are wider and samples are unevenly distributed across petro-groups. This data fits with geochemical results too. As for handmade vessels (Table 12), it is mostly the monochrome and red-black burnished ware (M/RBBW) that is responsible for the high petrographic variability of this varied group of containers. Indeed, when we exclude this ware from the calculations, the handmade vessels become much closer to the wheel-finished ones. Parameters that still suggest a much stronger variability are the high incidence of petro-loners, the low average number of samples per petro-group and the high Jaccard’s dissimilarity. By distinguishing the various wheel-finished wares (Table 12), we notice that the mass-produced bowls are the least variable for almost all the considered parameters. Further significant data emerge when we compare vessels sharing similar formal and functional features but differing in the forming procedures. For instance, kitchen wares can be invariably handmade or finished on the wheel, but this has no influence on the standardization degree of recipes, as both categories exhibit quite similar values.

**Table 11:** Values of the diversity parameters considered for the different ceramic wares and manufacturing techniques within each LC sub-phase.

		RICHNESS		EVENNESS			RICHNESS+EVENNESS	DISPARITY
		% petro-loners	Menhinick’s	Disparity in recipe abundance	Average nr of samples per petro-group	Pielou’s	Shannon’s	Jaccard’s dissimilarity %
**LC1-2**	handmade mass-produced bowls	60	1.79	20	/	0.96	1.33	/
	handmade plain ware	60	1.79	20	/	0.96	1.33	/
	handmade burnished ware	100	2.24	0	/	1	1.61	/
	handmade plain grit ware	50	1.5	25	/	0.95	1.04	/
**LC3-4**	handmade wares	65	3.84	11	2.33	0.97	2.81	66.67
	handmade—M/RBBW	41.67	2.67	15	2.33	0.96	2.2	66.67
	wheel-finished wares	5.19	1.37	22	9.12	0.82	2.04	55.83
**LC5**	handmade wares	51.72	3.34	21	4.67	0.89	2.57	72.22
	handmade—M/RBBW	33.33	1.63	16	2	0.96	1.33	66.67
	wheel-finished wares	4.54	1.7	19	3	0.95	1.98	55.16

**Table 12:** Values of the diversity parameters considered for the different handmade and wheel-finished ceramic wares of the LC3-4 and LC5.

			RICHNESS		EVENNESS			RICHNESS+EVENNESS
			% petro-loners	Menhinick’s	Disparity in petro-group abundance	Average nr of samples per petro-group	Pielou’s	Shannon’s
**LC3-4**	**Handmade**	M/RBBW	100	3	1	/	1	2.2
		kitchen ware	42.86	1.89	15	/	0.96	1.55
		light-colored ware	60	1.79	20	/	0.96	1.33
	**Wheel-finished**	kitchen ware	42.86	3.78	15	2	0.96	1.55
		light-colored fine ware	37.5	3.89	49	5	0.77	1.07
		red-slipped burnished ware	6.25	5.93	28	7.5	0.86	1.54
		mass-produced bowls	7.69	3.88	53	6	0.78	0.86
		chaff-tempered ware	17.65	4.5	23	4.67	0.92	1.66
**LC5**	**Handmade**	M/RBBW	56.52	3.33	18	3.33	0.89	2.31
		kitchen ware	33.33	1.63	16	2	0.95	0.56
	**Wheel-finished**	light-colored fine ware	27.27	1.8	19	2.66	0.93	1.67
		light-colored semi-fine ware	40	1.34	40	3	0.86	0.95
		mass-produced bowls	0	1.22	1	2	1	1.09

The variability indexes assessed for each ware (Table 12) allow us to nuance the trends obtained chemically. Consistently with chemical results, the handmade monochrome/red-black burnished wares are associated with kitchen wares as it concerns the high petrographic variability. Both handmade and wheel-finished kitchen wares show high percentages of petro-loners, high Pielou’s and Shannon’s indexes, a low disparity in petro-group abundance as well as a low average number of samples per petro-group. The wheel-finished red-slipped burnished ware, which has an intermediate chemical variability, exhibits the highest Menhinick’s index, but the lowest percentage of petro-loners, the highest average number of samples per petro-group and a relatively high disparity in abundance between the most and less represented petro-group. By contrast, the handmade light-colored ware, the wheel-finished chaff-tempered smoothed, and fine light-colored ware, which are chemically more homogeneous than the red-slipped burnished ware, have more loners, smaller group sizes, a generally higher Pielou’s index and a lower disparity in petro-group abundance, although their Menhinick’s and Shannon’s indexes still appear lower.

The average number of samples per petro-group and the Jaccard’s dissimilarity % were not calculated in cases of low number of samples and/or high incidence of petro-loners.

The average number of samples per petro-group and the Jaccard’s dissimiliraty % were not calculated in cases of low number of samples and/or high incidence of petro-loners.

In the final phase of the LC, the wheel-finished vessels still show a lower petrographic variability compared to the handmade ones (Table 11), but the difference is now less marked especially in terms of evenness. Among the handmade wares (Table 12), the monochrome and red-black burnished ware (M/RBBW) again exhibits the highest variability. If we exclude this ware from the calculations, the handmade vessels become even less variable than the wheel-finished ones in terms of Mehinick’s and Shannon’s indexes, while the incidence of petro-loners and Jaccard’s dissimilarity continue to suggest a higher variability. As for the various wheel-finished wares (Table 12), the mass-produced bowls still show the lowest petrographic richness, as in the previous phases, but evenness is now higher than in other wheel-finished vessels. Indeed, Pielou’s index provides higher values and thin sections are more evenly distributed across petrographic groups.

When we compare vessel categories that recur both in the LC3-4 and LC5, interesting diachronic trends emerge. Diversity indexes change differently through time according to forming techniques. The handmade production shows an unequivocal trend from the LC3-4 to LC5 towards a petrographic homogenization in terms of both richness and evenness, while the wheel-finished production tends to lose in homogeneity (Table 11) despite an increased use of rotating devices in LC5. With time the values of almost all diversity indexes increase and petro-group sizes decrease. As for mass-produced bowls (Table 12), although always more homogenous than other coeval wheel-finished wares, they do not show univocal trends when considered diachronically: their petrographic richness tends to decrease, while their petrographic evenness and disparity increases. Kitchen wares become instead petrographically more homogeneous even though by the LC5 they are exclusively fashioned by hand. The handmade monochrome/red-black burnished ware exhibits the highest variability within each period, but clearly tends towards a petrographic homogenization in the course of time, as revealed by the significant decrease in petro-loners and evenness by the final Late Chalcolithic phase. Finally, consistently with the chemical trends, the LC5 differs from the LC3-4 by the lower disparity in petrographic variability that separates the single wares (Table 12).

## Discussion and conclusions

The application of diversity statistics to geochemical and petrographic data sheds light on the craft organization of Arslantepe Late Chalcolithic pottery. All data suggest that the higher standardization of ceramic recipes is connected with the scale or rate of production rather than with the use of rotating devices. Mass-produced vessels, both the handmade ones (LC1-2 and partially in LC3-4) and the ones shaped on the wheel (partially LC3-4 and LC5), indeed display the lowest compositional variability within each period. A close relation between the emergence of serial production and the progressive homogenization of the *chaîne opératoires*, involving also a stronger selection of paste recipes, has been already identified in the Late Chalcolithic contexts from northern Mesopotamia and the Levant [30]. According to the CVs calculated on morphometric values of different types of wheel-finished and handmade vessels (Table 1), the increasing use of the wheel by the final Late Chalcolithic did not even perfectly match an increased standardization of vessel shapes [64, 69]. This evidence is not surprising: several ethnographic studies demonstrate that the forming technique does not usually affect the morphological variability of ceramic assemblages [27, 88]. This data has been recently questioned by Balossi Restelli [52: 488–489] at least concerning the LC3-4 mass-produced bowls, which provide progressively lower formal CVs throughout time as the implementation of rotational kinetic energy (RKE) increases. However, these figures still display a higher formal standardization than the LC5 mass-produced bowls, in which the use of RKE is further increased. At Arslantepe morphometric CVs do not even evidence clear differences between mass-produced bowls and other vessels [64, 69]. Thus, variations in the production rate affect the strategies of raw material supply and processing rather than vessel shape variability. Morphometric features might depend on many factors besides craft specialization and production rate, such as contexts of use, vessel sizes, levels of care and number of individuals involved in the production [101]. Hruby [101] interpreted for instance the high metrical variability of ceramics found in the Mycenaean palace of Nestor as the result of the high speed of production in a context intended for consumption by people of lower rank. This hypothesis could also fit the mass-produced bowls from Arslantepe that provided a clear evidence of negligence and time pressure along the manufacturing sequence (e.g. drying cracks, finger imprints, rough repairs, extended dark cores, black firing spots) [73]. Gosselain provides further clues to interpret the differences in variations between metrical and petro-chemical features observed in this case-study [102]. As opposed to raw material procurement and processing, procedures such as vessel shaping rely on an embodied knowledge acquired through learning networks and non-discursive cognitive processes, which leaves wider space for individual variance from models. Furthermore, the raw material and selection have the lowest visual impact on finished vessels and as such most closely reflect traditions of potters and changes in craft standardization. In any case, as argued by Kotsonas [24], standardization is a relative concept that can only be approached by comparing different vessel attributes (e.g. fabrics, shapes, dimensions, decorations).

During the LC3-4, the geochemical and petrographic variability is also influenced by the types of surface treatments and firing conditions. Within the wheel-finished productions, the red-slipped burnished ware has relatively variable raw materials and paste recipes, which are both widely used and never the result of random choices. This could indicate that they were realized in multiple but well-established production nuclei. This seems to corroborate previous petrographic and geochemical results [85], which indicated for this ware the use of distinct raw materials and paste preparation for open- and closed-shaped vessels. By contrast, although both wheel-finished and handmade non-mass-produced light-colored wares indicate the exploitation of relatively homogeneous clay sources (i.e. homogeneous geochemistry), the modes of processing them (e.g. tempering and mixing) did not follow fixed criteria. Kitchen wares, whether handmade or wheel-finished, are often the most heterogeneous just behind the handmade red-black/monochrome burnished ware, with which they sometimes share similar surface treatments and firing procedures. The affinity between these two classes of handmade vessels will further consolidate in the following LC5 phase, when both share exactly the same raw materials and paste recipes [84].

Among the various indexes applied in this paper the incidence of petrographic loners has repeatedly been shown to be an eloquent indicator of lower standardization. This result has twofold methodological outcomes: at the level of petrographic analysis of ceramic artifacts, we should as much as possible avoid forcing a grouping of thin sections in cases of insufficient common features; and at a more general level, we should dedicate more attention to what is outside of normality (deviant and variant types) among local assemblages, since local outliers best express the peak of diversity—in terms of both richness and disparity—that can be reached in a production place.

While issues related to taxonomic classifications have been extensively discussed in archaeology, above all concerning typological methods, they have not been exhaustively examined in the field of archaeometric applications. In grouping and interpreting archaeological artifacts based on chemical and mineralogical compositions, we should more often remember the words of Foucault in the preface of “The Order of Things: An Archaeology of the Human Sciences”: “there is nothing more tentative, nothing more empirical (superficially, at least) than the process of establishing an order among things […]. There is no similitude and no distinction, even for the wholly untrained perception, that is not the result of a precise operation and of the application of a preliminary criterion” [103: *xxi*]. From the Foucauldian perspective, taxonomic classifications, though providing a ground grid for the scientific study, present clear limitations as a result of a subjective reality representing only one among numerous alternative schemes.

Going back to our case study, different diachronic trends emerge among handmade and wheel-shaped vessels. The former univocally tend towards a higher standardization that reaches its peak in the final Late Chalcolithic phase, when economic centralization increases, the political and administrative power of the elites appears more pervasive, and food distribution became detached from the ritual sphere [45: 7–19]. The handmade red-black/monochrome burnished ware, which constantly exhibits the highest diversity within each period, is no exception to this trend. Nevertheless, in this case changes in the strategies of subsistence and mobility practices might have also played a significant role: the handmade red-black/monochrome burnished ware is commonly associated with mobile pastoral groups that gradually established themselves at, and possibly around, the site [104: 53, 105: 171]; from LC3-4 to LC5, as the sedentariness of these groups and their integration with the more sedentary components of the Malatya Plain communities increased, I believe that the areas exploited for the procurement of raw materials became closer and narrower and the resulting recipes more standardized [84]. This process continued and became more evident in the following Early Bronze Age 1 phase (3000–2800 BCE), when the exploitation of the Malatya metamorphics distributed over an area of 10 to 30 km south of the site drastically decreased in favor of the much closer Orduzu volcanics [84]. As for wheel-shaped vessels, the last Late Chalcolithic phase 5 marks a geochemical homogenization but a petrographic and dimensional diversification, which might suggest an increased standardization in the exploitation of clay sources but a decreased standardization in paste recipes and forming procedures. I would like to propose a hypothesis, which however needs further data to be verified, and namely that this might indicate a process of division within the operational sequence between people that procured the raw material and those dedicated to potting, that is to the subsequent production stages. During the LC5 period, the procurement of raw materials for the wheel-finished wares possibly occurred at a collective level according to a higher degree of interaction and co-operation. It is also possible that, compared to the past, the processing of raw materials and vessels’ shaping might have involved more individuals, who acted more independently and in more isolated ways from each other, and this would account for the increased metrical diversity within each morphological type. Another piece of evidence needs to be recalled here: the disappearance of potters’ marks in the LC5 period, marks that during the LC3-4 had allowed the producers to recognize their own vases in communal drying and firing areas, further corroborates the hypothesis of a reduced interaction among potters, and possibly the disintegration or reconfiguration of former communities of practices [64, 106]. The more LC5 centralized system conceivably exercised more control over the exploitation of resources rather than over other steps of the manufacturing sequence, which left wider space for individual choice and creativity. More generally at a macroscopic level, the pronounced labor division led to a reduced amount of types and wares that, however, differ more strongly from each other [52, 62, 64, 65]. In terms of diversity statistics, the general richness of ceramic assemblages decreases, but their disparity increases, which implies a strong morpho-functional specialization [64]. Peculiar to the LC5 is also the reduced gap between the diversity indexes calculated on the petrographic and geochemical data of each ware. Unlike in the LC3-4, the combination of technological and functional features represented by each ware do not correspond to a specific standardization level in raw materials and paste recipes. This set of results prompts us to reconsider the direct relationships often simplistically established between standardization and specialization. As we can clearly observe at Arslantepe, the specialization of tasks within the *chaîne opératoire* that marks the end of the Late Chalcolithic period does not coincide with an increased standardization but, on the contrary, with a higher variability of both technical procedures and end products. Further south of Arslantepe, in the northern Mesopotamian sites of Hamoukar and Tell Brak (Khabur basin), diachronic trends towards standardization appear more univocal and visible through an increased uniformity both at a typological and technological level [29]. The higher degree of urbanization reached in those areas [107] might have created a spatial and social conjunctive tissue enhancing the transmission and sharing of models and practices between vessel makers.

At Arslantepe, the mass-produced bowls illustrate especially well the shift from communal to more centralized—but possibly less integrated—potting practices in relation with increased social complexity, production rate and rotational speed of the wheel. Indeed, the diversity parameters of the mass-produced bowls indicate a clear trend towards the use of a reduced range of recipes, all equally well-established and markedly differing from each other. This is accompanied by a progressive diversification of manufacturing procedures, shapes and sizes [64, 69, 73].

This work questioned the assumed unilinear correspondence between the increase in craft standardization, the use of the rotational kinetic energy and the emergence of economic centralization. The results obtained encourage us to explore artifacts’ standardization through a threefold scheme of diversity in relation to various compositional, technological, typological and morphometric features in order to account for the complexity of the social organization of the pottery production. By de-structuralizing the concepts of diversity and operational sequence we can better understand the modalities and causes of standardized behaviors and gestures [33] and gain significant clues about the control over natural resources and labor division exercised by centralized political and economic systems. In the future, standardization studies should dedicate more attention to assessing and comparing the variability of non-metric data such as the petrographic and typological classifications, thus focusing on the different forms and degrees of specialization. As this paper clearly demonstrates, there is no single notion of specialization and standardization, for which we have to think plural. The present approach has shown to be suited to diachronic investigations at an intra-site level and seems appropriate in cases of variegated artifact assemblages and geological landscapes. However, petro-loners as well as the indexes used to assess the petrographic evenness could also be theoretically employed for inter-site comparisons as they are not influenced by the geological variability. The results allowed us to speculate on key aspects of socio-economic relationships and modes of labor organization in the crucial time of state formation. On this basis, an enlargement of samples and a further statistical elaboration are planned to test the method on different archaeological and geological contexts and support inter-site comparisons of pottery craft standardization. Ultimately, this paper intends to provide food for transdisciplinary thoughts on the fluid concept of diversity and to question human schemes of categorization and hierarchization of things.

## Supporting information

S1 Table Series of variation matrixes calculated on: LC sub-phases, ceramic wares and assemblages fashioned with different techniques and/or production rates. (XLSX)

S2 Table Calculations of Mehinik’s, Pielou’s and Shannon’s indexes. (XLSX)

S3 Table Calculations of Jaccard’s dissimilarity indexes based on presence/absence variables. (XLSX)
